# Pigment–Structure Leaf Syndromes in Poinsettia (*Euphorbia pulcherrima* Willd. ex Klotzsch) Alter Chloroplast Ultrastructure, Biochemical Profiling, and Photosynthetic Quantum Yields

**DOI:** 10.1021/acsomega.6c01933

**Published:** 2026-05-04

**Authors:** Renan Falcioni, Werner Camargos Antunes, José Alexandre M. Demattê, Marcos Rafael Nanni

**Affiliations:** † Graduate Program in Agronomy, 42487 State University of Maringá, Av. Colombo, 5790, Maringá 87020−900, Paraná, Brazil; ‡ Department of Biology, State University of Maringá, Av. Colombo, 5790, Maringá 87020−900, Paraná, Brazil; § Department of Soil Science, Luiz de Queiroz College of Agriculture, University of São Paulo, Av. Pádua Dias, 11, Piracicaba 13418−260, São Paulo, Brazil

## Abstract

Colored bracts and leaves are widespread in plants; however, how their pigments and structures jointly control the fate of absorbed light is poorly understood. We used contrasting albino and red Poinsettia (*Euphorbia pulcherrima* Willd. ex Klotzsch) phenotypes to test how selection for extreme color morphs reshapes chloroplast ultrastructure, photosynthetic quantum yield, and the spectral signatures of colored canopies. We combined hyperspectral and thermal imaging, pigment, biochemical and antioxidant profiling, gas exchange and chlorophyll a fluorescence, and light and electron microscopy across four tissues: albino bracts, green leaves of albino plants, red bracts, and green leaves of red plants. These tissues form four distinct “pigment–structure leaf syndromes” that decouple light capture from carbon assimilation. Albino and anthocyanin-rich red bracts retained functional photosystem II (PSII) but contained very few, weakly stacked chloroplasts, showed much lower stomatal and mesophyll conductance and carboxylative capacity, and behaved as photoprotective, antioxidant sinks with almost zero net CO_2_ uptake. By contrast, the companion green leaves—especially those of red plants, developed a thick mesophyll with large intercellular airspaces and numerous grana-rich chloroplasts, reached the highest maximum Rubisco carboxylation, electron transport, and triose-phosphate-use capacities, and achieved the highest absorbed-light quantum yield of CO_2_ assimilation, thereby compensating for albino and red bracts at the module and whole-plant scale. Across tissues, absorptance and PSII quantum yield alone failed to predict CO_2_-assimilation quantum yield, which instead tracked chloroplast number, thylakoid investment, and CO_2_ conductance. Full-range ultraviolet-to-shortwave-infrared spectra and multivariate analyses captured these syndromes as distinct optical fingerprints, providing a mechanistic framework for interpreting hyperspectral signals from canopies containing nongreen or variegated organs.

## Graphical abstract

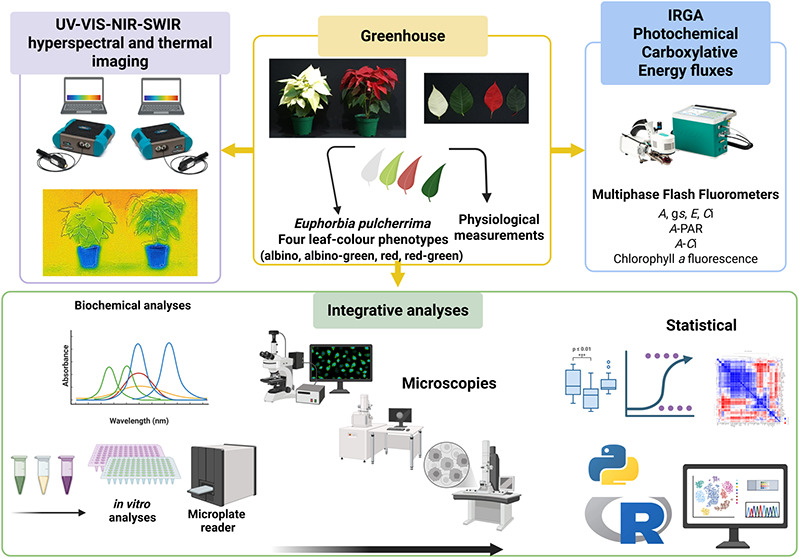

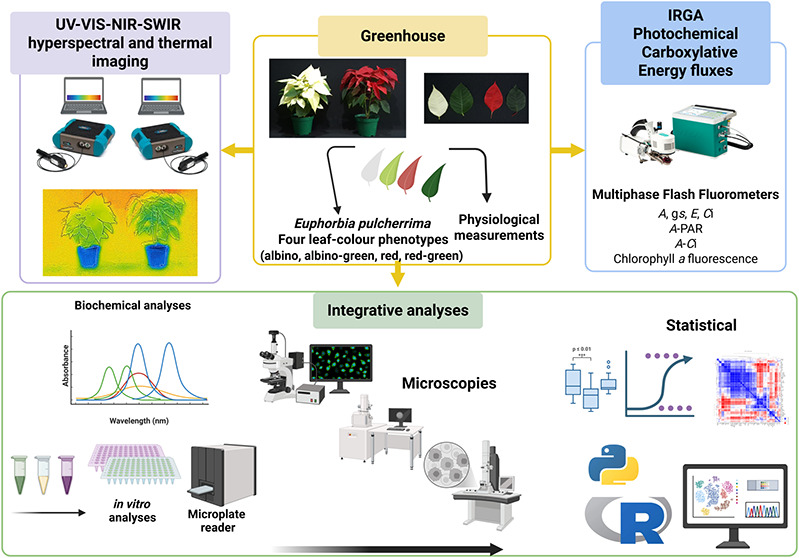

## 1 Introduction

Leaves are the main interfaces for photon capture and carbon assimilation. However, many plants simultaneously deploy conspicuously colored or chlorophyll-deficient organs, including bracts, variegated sectors, or multicolored leaves.^1^ These tissues create a persistent physiological paradox: canopy optical signals can change markedly, while net CO_2_ gain may remain unchanged or even decline because color is not a direct readout of the capacity to convert absorbed light into carbon assimilation. The mechanistic challenge is therefore to explain when and why light capture and photochemical activity become uncoupled from the quantum yield of CO_2_ assimilation, particularly in canopies that mix green foliage with nongreen display tissues.

Nongreen organs typically contain little chlorophyll, show altered internal anatomy, and depend on adjacent green tissues for energy and carboxylative yields, yet they can retain measurable photochemistry and absorb substantial radiation.^2,3^ Whether such organs behave as inefficient photosynthetic surfaces or as specialized sinks hinges on how their pigment composition and structure control the fate of absorbed photons. In mechanistic terms, coloration in these tissues results from the combined action of chloroplastidic pigments (chlorophylls and carotenoids organized in thylakoid membranes), extra-chloroplastidic screens (anthocyanins, flavonoids, and phenolics, often epidermal or vacuolar), and stomata that reshape the internal light field and redox environment.^3–5^

Recent work has refined how the PSII antenna system dynamically balances light harvesting and photoprotection,^6,7^ while emerging structural perspectives emphasize that grana architecture and thylakoid investment constrain how much electron-transport capacity can be deployed per chloroplast and per mesophyll cell.^8,9^ In colored or chlorophyll-deficient tissues, these determinants often covary with mesophyll anatomy and CO_2_ diffusion pathways, so that the limiting step shifts from photon supply to CO_2_ delivery and metabolic sink strength. Under such sink limitation, PSII can remain photochemically competent, yet the products of photochemistry (ATP and NADPH) cannot be efficiently used for net carboxylation, forcing excitation into regulated heat dissipation and alternative sinks.^10–12^ This covariation motivates a syndromic view: pigments, mesophyll architecture, and chloroplast ultrastructure form integrated “pigment–structure syndromes” that govern energy partitioning and, ultimately, the photosynthetic yield.

Hyperspectral sensing and chlorophyll fluorescence provide complementary, nondestructive routes to diagnose these pigment–structure leaf syndromes. UV–VIS–NIR–SWIR reflectance/transmittance spectra integrate pigment absorption, mesophyll scattering, and water/structural features, while longwave infrared (LWIR) thermal imaging provides a complementary readout of energy dissipation.^13–16^ Fluorescence yields and OJIP/JIP descriptors report PSII excitation pressure and alternative energy pathways, offering functional constraints that reflectance alone cannot provide.^6,13^ Electron microscopy, in turn, resolves the thylakoid “hardware” that sets an upper bound on electron transport and ATP/NADPH supply and reveals how the chloroplast architecture is remodeled in colored and chlorophyll-deficient organs.^12,17^ Despite these advances, most studies still link only fragments of the pathway from pigment composition to whole-leaf function, often across different species or genetic backgrounds, which makes it difficult to attribute optical phenotypes to specific structural and physiological mechanisms.^14,16,18,19^ What remains particularly underresolved is an integrated within-system mapping from pigments and radiative transfer to chloroplast ultrastructure, CO_2_ diffusion, and the absorbed-light-based quantum yield of CO_2_ assimilation (αCO_2_,abs). This still leads to the unresolved question of how pigment–structure syndromes affect the quantum yield of photosynthesis in photosynthetic plant tissues.

Poinsettia (*Euphorbia pulcherrima* Willd. ex Klotzsch) is a suitable model to address this gap because extreme color morphs coexist with companion green leaves within the same canopy architecture. During bract development, chlorophyll declines while anthocyanins accumulate, generating nongreen display tissues that remain exposed to high irradiance.^4^ The key unresolved issue is whether these color morphs reveal generalizable principles about how pigment screens and chloroplast investment jointly determine the coupling between photochemistry and carbon gain and whether such mechanisms can be diagnosed from hyperspectral and multivariate signatures in mixed canopies.

Here, we exploit contrasting albino and red poinsettia phenotypes that generate large shifts in extra-chloroplastidic screening and chloroplast investment within a comparable canopy module. We analyze four tissues (albino bracts, green leaves of albino plants, red bracts, and green leaves of red plants) to test whether: (H1) colored bracts form low-yield, photoprotective syndromes in which limited chloroplast “hardware” and CO_2_-use capacity divert absorbed energy away from net carboxylation; (H2) companion green leaves compensate via coordinated increases in CO_2_ diffusion, biochemical sink strength, and chloroplast ultrastructure; and (H3) these syndromes generate stable hyperspectral and multivariate fingerprints that enable mechanistic interpretation of mixed colored canopies.

## 2 Materials and Methods

### 2.1 Plant Material and Experimental Design

Poinsettia (*E. pulcherrima* Willd. ex Klotzsch) plants with contrasting bract colors were grown in a greenhouse at the Botanic Garden, State University of Maringá (Maringá, Paraná, Brazil). We used two commercial cultivars from the Christmas Feelings series: Christmas Feelings White (albino-type bracts) and Christmas Feelings Red (anthocyanin-rich red bracts). Rooted cuttings were transplanted into 2 L plastic pots filled with a peat-based commercial substrate (MecPlant, Telêmaco Borba, Brazil) and grown under natural daylight only. Plants were irrigated daily to drainage with Hoagland nutrient solution (pH 5.5). During cultivation, daytime and night-time air temperatures were typically 24–30 °C and 18–22 °C, respectively, and relative humidity ranged from 60% to 80%.

At full bract coloration, four tissue types were sampled: albino bracts (AB), green leaves of albino plants (AG), red bracts (RB), and green leaves of red plants (RG). For each tissue, fully expanded organs were collected from the midcanopy of ten independent plants (*n* = 10) and allocated to biochemical, optical, physiological, and anatomical analyses. The experiment followed a completely randomized design with tissue type as the fixed factor.

### 2.2 Hyperspectral and Thermal Optical Leaf Properties

Leaf reflectance (*R*) and transmittance (*T*) were measured simultaneously on intact, attached bracts and leaves using two calibrated FieldSpec 3 spectroradiometers (ASD Inc., Longmont, CO, USA) coupled to 10 mm Contact PlantProbe leaf clips. Each instrument covered 350–2500 nm (UV–VIS–NIR, 350–1000 nm; SWIR1, 1000–1800 nm; SWIR2, 1800–2500 nm) (Falcioni et al.)^19,20^ . One PlantProbe (lamp on) was placed on the adaxial surface to record reflected light, while a second PlantProbe (lamp off) was aligned on the abaxial surface to record transmitted light along the same optical path.^19^ Spectra were acquired at 1 nm intervals after wavelength-dependent calibration against a Spectralon white reference (Labsphere Inc., North Sutton, NH, USA). Leaf absorption (Abs; defined here as the fraction of incident radiation absorbed) was calculated as Abs = 1 – (*R* + *T*). This method is more robust than an integrating sphere, as it is fast and simultaneously measures reflectance, transmittance, and absorption in vivo, without interference or the need to reposition the leaves.^17,19,20^ For hyperspectral measurements, four spectra were acquired per organ per plant and averaged to yield one spectrum per plant (*n* = 10 biological replicates per tissue) for inferential statistics; the full set of spectra (*n* = 40 per tissue) was used for PCA visualization.^21^

Longwave infrared thermal images were acquired with a radiometric FLIR Vue Pro camera (FLIR Systems, Danderyd, Sweden; 7.5–13.5 μm) at a fixed distance from the canopy. Mean surface temperatures were extracted from regions of interest corresponding to bracts and true leaves using the manufacturer’s software.

### 2.3 Biochemical Analyses

From each plant, discs (≈1 cm^2^) were collected from bracts or leaves and pigments extracted following the chloroform:methanol protocol of Gitelson and Solovchenko (2018).^21^ Samples were homogenized in chloroform:methanol (2:1, v/v) containing CaCO_3_, and phase separation was induced by adding water (20% of final volume) followed by centrifugation (15,000 rpm, 5 min). The chloroform-rich phase (chlorophylls and carotenoids) and the methanol–water phase (extra-chloroplastidic pigments) were transferred to quartz 96-well microplates (200 μL well^–1^) and read in a UV–VIS microplate reader (Biochrom Asys UVM-340; Biochrom Ltd., Cambridge, U.K.). Chlorophyll/carotenoid absorbance was recorded at 665, 652, and 470 nm and converted to chlorophyll a, chlorophyll b, total chlorophyll (Chla+b), and total carotenoids using equations described by Gitelson and Solovchenko (2018),^21^ expressed on an area basis (mg m^–2^) and mass basis (mg g^–1^ dry mass), with pathlength correction for microplates.

Flavonoids were quantified in the methanol–water phase at 358 nm, and anthocyanins were quantified at 530 nm after acidification to 0.1% (v/v) HCl, using the coefficients described by Gitelson and Solovchenko (2018)^21^ with cyanidin 3-glucoside as reference. Total soluble phenolics were determined by Folin–Ciocalteu and expressed as milligrams of gallic acid equivalents (GAE) g^–1^ dry mass. Antioxidant capacity was assessed by DPPH radical scavenging and expressed as percentage quenching relative to reagent blanks.

### 2.4 Cell-Wall Composition Analyses

Protein-free cell-wall (PFCW) fractions were prepared from freeze-dried, ground poinsettia tissues following Moreira-Vilar et al.^22^ and Falcioni et al.^19,20^ Approximately 150 mg of dry powder from each sample was placed in 2 mL microtubes and subjected to sequential washes to remove soluble metabolites and proteins: five washes with 50 mM potassium phosphate buffer (pH 7.0), five with 50 mM phosphate buffer containing Triton X-100, four with 1 M NaCl, four with distilled water, and three with acetone. After each wash, the samples were centrifuged (≈15,000 rpm, 3 min). The final pellets were dried at 60 °C for 24 h and considered to be PFCW (protein-free cell wall).

#### 2.4.1 Lignin Quantification

Lignin in the PFCW was quantified via the acetyl bromide soluble lignin assay (ABSL) according to Moreira-Vilar et al.,^22^ with microplate adaptation as described by Falcioni et al.^23^ For each sample, 10 mg of PFCW was transferred to microtubes and incubated with 130 μL of a freshly prepared acetyl bromide solution (25% v/v in glacial acetic acid) at 70 °C for 30 min. After cooling on ice, 0.24 mL of 2 M NaOH, 0.02 mL of 5 M hydroxylamine-HCl, and 1.6 mL of glacial acetic acid were added to complete lignin solubilization. The tubes were subsequently centrifuged (≈1400*g*, 5 min), and 200 μL of the supernatant was dispensed into UV-transparent 96-well plates. The absorbance at 280 nm was read in a microplate reader, and the lignin concentration was calculated from an alkali lignin calibration curve using an extinction coefficient ε = 22.1 L g^–1^ cm^–1^. The results are expressed as milligrams of lignin g^–1^ of PFCW.

#### 2.4.2 Cellulose Quantification

Cellulose was quantified in the same PFCW using the anthrone colorimetric assay adapted for microplates.^23^ For each sample, 10 mg of PFCW was incubated with an acetic acid/nitric acid mixture at 70 °C (≈1 h) to remove noncellulosic polysaccharides, washed thoroughly with distilled water, and resuspended in concentrated H_2_SO_4_. Aliquots of this suspension were reacted with freshly prepared anthrone reagent in H_2_SO_4_, incubated in a water bath, and cooled on ice, and 200 μL was transferred to 96-well plates. The absorbance was read at 620 nm in a microplate reader, and the cellulose content was calculated from a glucose standard curve and expressed as glucose equivalents (mg glucose g^–1^ PFCW).

### 2.5 Gas-Exchange Measurements

Gas exchange was measured on fully expanded, healthy bracts and true leaves from the midcanopy at full bract coloration using an infrared gas analyzer (LI-6800; LI-COR Biosciences, Lincoln, NE, USA) coupled to a Multiphase Flash fluorometer (LI-6800–01). Net CO_2_ assimilation (A), intercellular CO_2_ concentration (*C*_i_), stomatal conductance to water vapor (*g*_s_), and transpiration (*E*) were recorded, with fluorescence acquired simultaneously during the A–PAR, *A*–*C*_i_, and fluorescence curves. For each tissue type, gas exchange and concurrent fluorescence were measured on four organs per plant, and values were averaged to produce one plant mean used as the biological replicate (*n* = 10).

#### 2.5.1 A–PAR Response Curves

A–PAR curves were obtained by stepping photosynthetically active radiation (PAR) from saturating irradiance to darkness at 2500, 2000, 1800, 1500, 1200, 1000, 800, 600, 400, 300, 200, 150, 100, 75, 50, 25, and 0 μmol photons m^–2^ s^–1^, with 50–70 s per step. Measurements were conducted at 25 °C leaf temperature, 400 μmol mol^–1^ reference CO_2_, ∼60% relative humidity, 700 μmol s^–1^ flow, 10,000 rpm fan speed, and a red/blue actinic ratio of 90:10. At each step, variables were logged after reaching a steady state.

Light-response curves were fitted with a nonrectangular hyperbola; when net assimilation was near zero across most irradiances, a constrained low-light linear fit was applied. From fitted curves, we derived dark respiration (*R*_d_), the apparent quantum yield (α), maximum net assimilation (PNmax), the light compensation point (LCP; A = 0), and the light saturation point (LSP; PAR at 90% of PNmax). Net assimilation at 1500 μmol of photons m^–2^ s^–1^ (A_1500_) was taken directly from the measured curve. Intrinsic water-use efficiency was computed as A_1500_/*g*_s_ at 1500 μmol of photons m^–2^ s^–1^. Absorbed-light-based quantum yield (αCO_2_,abs) was calculated using the absorbed photon flux derived from leaf absorption measurements (Section 2.2). Where the LI-6800 output provided the electron-transport rate (*J*), ATP and NADPH production rates were estimated from linear electron transport and reported at 1500 μmol of photons m^–2^ s^–1^.

#### 2.5.2 *A*–*C*_i_ Response Curves and Parametrization

*A*–*C*_i_ curves were measured on the same tissues under saturating PAR (1500 μmol photons m^–2^ s^–1^) by stepping reference CO_2_ (*C*_a_) at 400, 300, 200, 100, 50, 400, 500, 600, 800, 1000, 1200, 1500, 1800, and 2000 μmol mol^–1^, with 70–90 s per step. At each step, *A*, *C*_i_, fluorescence parameters, leaf temperature, and *g*_s_ were recorded after stabilization.

Curves were fitted with a three-limitation Farquhar–von Caemmerer–Berry model to estimate Vcmax, Jmax, TPU, and day respiration (*R*_d_*), using temperature-corrected kinetic constants following standard parametrizations.^24–26^ Chloroplastic CO_2_ concentration (*C*_c_) and mesophyll conductance (*g*_m_) were obtained by numerical inversion of the fitted model to solve *C*_c_ for each measured (*A*, *C*_i_) point, followed by calculation of mesophyll conductance as $g_{m}=\frac{A}{C_{i}-C_{c}}$ . The net CO_2_ compensation point (Γ) and CO_2_ saturation point (*C*_iSAT_; 90% of fitted *A*_max_ per *A*–*C*_i_ curve) were determined from the fitted curves. ATP and NADPH requirements for CO_2_ assimilation were estimated at *C*_i_SAT assuming 3 ATP and 2 NADPH per mol of CO_2_ were fixed. Mesophyll conductance (*g*_m_) was interpreted only for points with *A* > 0; for tissues with near-zero or negative *A*, *g*_m_ estimates were adjusted for nonlinear models.

#### 2.5.3 Fast Chlorophyll Fluorescence Induction (OJIP) and JIP Test

Fast fluorescence induction kinetics (OJIP) were recorded using the LI-6800 system after overnight dark adaptation. Measurements were performed in a 2 cm^2^ chamber at 400 μmol mol^–1^ CO_2_, 75% relative humidity, and 10,000 rpm fan speed, using a 1 s saturating pulse (15,000 μmol m^–2^ s^–1^; 625 nm). Transients were sampled at high temporal resolution, and JIP-test parameters were derived using Biolyzer v4.0 (Laboratory of Bioenergetics, University of Geneva).^27–30^ Phenomenological energy-flux (pipeline) diagrams were constructed following the approach of Sitko et al.^31^

#### 2.5.4 Modulated Fluorescence during A–PAR Measurements

Chlorophyll fluorescence was measured on the same tissues during the A–PAR protocol using the Multiphase Flash procedure. After dark adaptation, *F*_0_ and *F*_m_ were determined, and under actinic light, *F*_s_, *F*_m_′, and *F*_0_′ were obtained at each PAR step with saturating pulses. Standard parameters were calculated (*F*_v_/*F*_m_, *F*_v_′/*F*_m_′, ΦPSII, NPQ, qP, qN, and qL). The quantum yield of CO_2_ assimilation per incident photon was computed as ΦCO_2_ = A/PAR. All fluorescence parameters reported in Table S1 correspond to 1500 μmol photons m^–2^ s^–1^, matching the high-light point used for gas-exchange comparisons.^24,32–35^

### 2.6 Microscopic Sample Preparation and Analyses

#### 2.6.1 Sample Preparation

Bract and leaf tissues (approximately 1 mm^2^) from albino bracts, green leaves of albino plants, red bracts, and green leaves of red plants were processed for light microscopy, scanning electron microscopy (SEM), and transmission electron microscopy (TEM). Samples were fixed in modified Karnovsky’s fixative (2.5% glutaraldehyde and 2% paraformaldehyde in 0.05 M sodium cacodylate buffer, pH 7.2).^36^ After fixation, tissues were postfixed for 6 h in 1% osmium tetroxide with 1.6% potassium ferrocyanide in the same buffer, contrasted overnight in bloc with 0.5% uranyl acetate, and dehydrated through an acetone series (30–100%, with three changes at 100%). Dehydrated samples were either prepared for SEM (see below) or embedded in Spurr low-viscosity epoxy resin for sectioning. Resin blocks were sectioned on an ultramicrotome (MTX Powertome X, Boeckeler Instruments) to obtain semithin (1.5 μm) and ultrathin (70 nm) sections using glass and diamond knives, respectively.

#### 2.6.2 Optical Microscopy Analyses

Semithin sections (1.5 μm) were stained with 1% toluidine blue in borax buffer for 30 s on a heating plate (70 °C) and imaged using a Leica ICC50 light microscope. Total leaf thickness, mesophyll thickness, and adaxial and abaxial epidermal thicknesses were quantified from calibrated images. In addition, fresh transverse sections were produced with a vibrating microtome (Leica VT1200 S) to visualize mesophyll organization and the distribution of chloroplastidic and extra-chloroplastidic pigments and were examined immediately after mounting in slides.

#### 2.6.3 Scanning Electron Microscopy Analyses

For SEM, dehydrated samples were critical-point dried (CPD-300, Bal-Tec), mounted on aluminum stubs, and gold-coated (MED010, Bal-Tec; 50 mA for 150 s). Micrographs were acquired on an FEI Quanta 250 SEM instrument at 20 kV. Stomatal density and trichome/papilla features were quantified from digital images.

#### 2.6.4 Transmission Electron Microscopy Analyses

Ultrathin sections (70 nm) were collected on 300-mesh copper grids and contrasted with 3% uranyl acetate for 40 min followed by lead citrate for 8 min.^37^ Observations were performed on a JEOL JEM-1400 TEM instrument operated at 80 kV and equipped with a digital camera. Ultrastructural features, including cell walls, chloroplasts and thylakoid membranes, plastoglobules, mitochondria, peroxisomes, vacuoles, and endomembrane structures, were examined and imaged.

#### 2.6.5 Quantification of Structures and Ultrastructures

Quantitative anatomical and ultrastructural traits were extracted from OM, SEM, and TEM images using ImageJ and Image-Pro Plus, complemented by custom Python routines for measurement handling. Traits included epidermal cell density, stomatal density and stomatal index, trichome/papilla density, mesophyll and epidermal thickness components, chloroplast area and number per mesophyll cell, thylakoid lamellae per granum, grana thickness, and derived ratios linking thylakoid investment to chloroplast abundance.

### 2.7 Statistical Analyses

#### 2.7.1 Univariate Analyses

Data homogeneity was assessed via Bartlett’s test for all variables.^38^ Quantitative data were analyzed by one-way analysis of variance (ANOVA) and presented as means ± SE. Statistical significance was determined at *p* < 0.01, and Tukey’s post hoc test was applied. When appropriate, Pearson’s correlation and cluster heatmaps were used to evaluate potential associations among variables. All the statistical analyses were performed via Statistica 12.0 (StatSoft Inc., Tulsa, Oklahoma, USA), SigmaPlot 12.0 (Systat, California, USA), R^39^, and custom Python (Python version 3.10) scripts for curve fitting, hyperspectral processing, and data visualization.

#### 2.7.2 Multivariate Analysis

To test overall tissue effects on correlated trait sets, MANOVA was applied prior to principal component analysis (PCA). PCA was used to reduce dimensionality and specifically to interpret coordinated variation underpinning the absorbed-light-based photosynthetic quantum yield (αCO_2_,abs) across AB, AG, RB, and RG tissues. PC1 and PC2 were retained for visualization, and tissue differences in mean scores were tested by one-way ANOVA with Tukey’s post hoc test (*p* < 0.01).^38^ For wavelength-by-wavelength comparisons of hyperspectral data, a more conservative threshold (*p* < 0.001) was adopted to reduce false positives across many wavelengths. Prior to PCA, variables were mean-centered and scaled to unit variance (*z*-scores). Trait-group contributions to each principal component were derived from PCA loadings/eigenvectors for biochemical composition, structure/ultrastructure, A–PAR traits, *A*–*C*_i_ traits, chlorophyll a fluorescence energy partitioning, JIP-test parameters, and phenomenological energy fluxes.^40^ Multivariate analyses and graphics were produced in R^39^ and Python (scikit-learn), with figure assembly in SigmaPlot, Python, and CorelDraw 2020.

## 3 Results

### 3.1 Visual Phenotypes and Pigment–Antioxidant Profiles

Four distinct tissue phenotypes were readily distinguished at full bract and leaf colorations (Figure 1a). Albino plants produced cream-white bracts (“albino bracts”; AB) subtended by pale-green foliage (“green leaves of albino plants”; AG), whereas red plants developed deep-red bracts (“red bracts”; RB) above dark-green foliage (“green leaves of red plants”; RG). Leaf shape was comparable between cultivars, but leaves from red plants appeared visibly darker than those from albino plants (Figure 1 inset).

**Figure 1:**
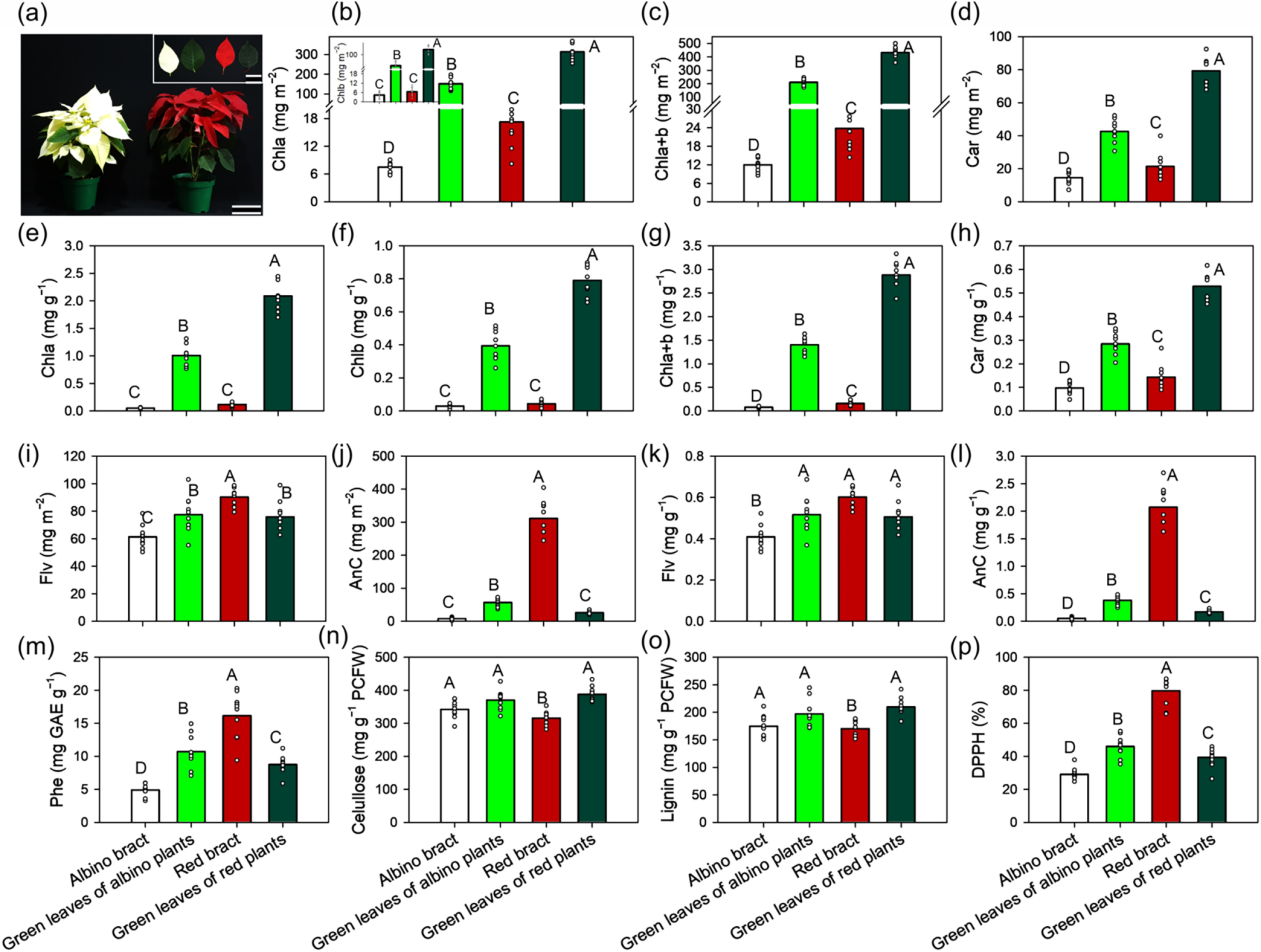
Poinsettia (*E. pulcherrima* Willd. ex Klotzsch) color phenotypes used to probe links between biochemical and pigment composition, leaf structure and ultrastructure, and photosynthetic function. (a) Left, albino-type plant with chlorophyll-deficient cream bracts and green leaves; right, red-type plant with anthocyanin-rich red bracts and green leaves. Inset (left to right): albino bract (AB), green leaves of albino plants (AG), red bract (RB), and green leaves of red plants (RG). Scale bars: 10 cm (whole plants) and 2 cm (isolated bracts/leaves). (b–p) Pigments, phenolic and cell-wall composition of poinsettia tissues. (b–d) Area-based contents of chlorophyll a (Chla), chlorophyll b (Chlb), total chlorophyll (Chla+b), and carotenoids (Car) (mg/m^2^). (e–h) Mass-based contents of Chla, Chlb, Chla+b, and Car (mg g^–1^ dry mass). (i–j) Area-based contents of flavonoids (Flv) and anthocyanins (AnC) (mg m^–2^). (k–l) Mass-based contents of Flv and AnC (mg g^–1^ dry mass). (m) Total phenolics (Phe; mg gallic acid equivalents (GAE) g^–1^ dry mass). (n–o) Cellulose and lignin (mg g^–1^ protein-free cell wall (PFCW)). (p) DPPH radical-scavenging capacity (%). The bars represent the means, and the symbols represent individual biological samples of albino bracts, green leaves of albino plants, red bracts, and green leaves of red plants. Different uppercase letters indicate significant differences among tissues (Tukey’s test, *p* < 0.01). Means ± SE (*n* = 10). PFCW, protein-free cell wall; DPPH, 2,2-diphenyl-1-picrylhydrazyl.

The pigment composition diverged sharply among tissues (Figure 1b–h). Chlorophylls (Chla, Chlb, and Chla+b) and carotenoids were concentrated in green leaves and strongly depleted in both bract types, consistent with a functional separation between photosynthetic and display tissues. On a leaf-area basis, RG contained ∼36-fold more Chla+b than AB and ∼18-fold more than RB, with AG showing intermediate enrichment (Figure 1c). The same ranking held on a dry-mass basis, with RG reaching ∼2.9 mg g^–1^ Chla+b while both bract types remained <0.2 mg g^–1^ (Figure 1g), reinforcing that bract pigmentation is not underpinned by chlorophyll investment.

In contrast, red-associated antioxidant traits tracked the pigmentation gradient (Figure 1i–p). Anthocyanins and total phenolics peaked in RB, which also exhibited the highest DPPH scavenging activity, whereas AB consistently showed the lowest values. RB accumulated ∼42-fold more anthocyanin per unit dry mass than AB, and anthocyanin content was tightly associated with DPPH activity across samples (*r* ≈ 0.93) (Figure 1l,p). Flavonoids varied less significantly than anthocyanins, suggesting that the dominant biochemical shift distinguishing RB is a strong commitment to vacuolar anthocyanin/phenolic enrichment rather than a broad increase in all phenylpropanoids. Cellulose and lignin contents were largely conserved across organs, yet RB showed a significant reduction in both structural polymers relative to those of AB and the green leaves (Figure 1n,o). Together, these data support a mechanistic division of labor in which green leaves maximize chlorophyll–carotenoid capacity for photochemistry, while red bracts adopt a pigment-based optical and antioxidant strategy that is compatible with low chlorophyll content (Figure 1a–p).

### 3.2 Leaf Cross-Sectional Anatomy and Hyperspectral Optical Traits

Cross sections showed coordinated anatomical differences among tissues (Figure S1). AB exhibited a relatively thin lamina with weak palisade differentiation and a loose spongy mesophyll, while AG developed a more distinct palisade and a denser mesophyll. RB were anatomically the simplest tissues, combining a very thin mesophyll with reduced intercellular airspace, whereas RG presented the most developed architecture, with a thick lamina, elongated palisade cells, and an extended spongy mesophyll. Quantitatively, adaxial and abaxial epidermal thicknesses were greatest in AG and lowest in RB (Figure S1m–o). Mesophyll thickness ranged from ∼70 μm (RB) to ∼220 μm (RG), with AB and AG intermediate (∼88–120 μm), and total thickness increased from ∼84 μm (RB) to ∼243 μm (RG), with AB and AG at ∼142 and ∼117 μm, respectively (Figure S1p). All anatomical traits differed significantly among tissues (Tukey’s test, *p* < 0.01), indicating that the visible phenotypes are embedded in distinct structural states rather than superficial color differences. Complementary SEM micrographs of epidermal surfaces are provided in Figures S2 and S3.

Hyperspectral measurements captured how these pigment–anatomy combinations shaped the optical behavior across 350–2500 nm (Figure 2a–c). In the visible region, AB were highly reflective and transmissive (at 550 nm, *R* ≈ 63% and *T* ≈ 27%), resulting in low absorption (≈10%), whereas green leaves showed the opposite behavior, consistent with chlorophyll-rich tissues (at 550 nm, AG and RG reflected ∼15% and ∼9% but absorbed ≈72% and ≈84%, respectively). RB exhibited the strongest green-band absorption (≈96% at 550 nm; reflectance ≈ 4%), consistent with dense accumulation of vacuolar red pigments. At 680 nm, AG and RG absorbed >90% of incident red light, whereas AB and RB still reflected ∼40% and transmitted ∼20%, indicating that, despite their strong visible coloration, bracts remain comparatively inefficient at red-light capture by chlorophyll-bearing complexes. In the NIR (≈800 nm), all tissues showed high reflectance (≈45–63%) and transmittance (≈31–44%) with low absorption (<12%), while in SWIR1 and SWIR2 (1650 and 2200 nm), green leaves transmitted more light (≈45–51%) than bracts (≈26–32%), consistent with differences in internal optical path lengths shaped by mesophyll architecture and water-related absorption features. Tissue effects on reflectance, absorption, and transmittance were strongly supported by ANOVA (*F* = 541.54, 48.87, and 312.78, respectively; *p* < 0.01).

**Figure 2:**
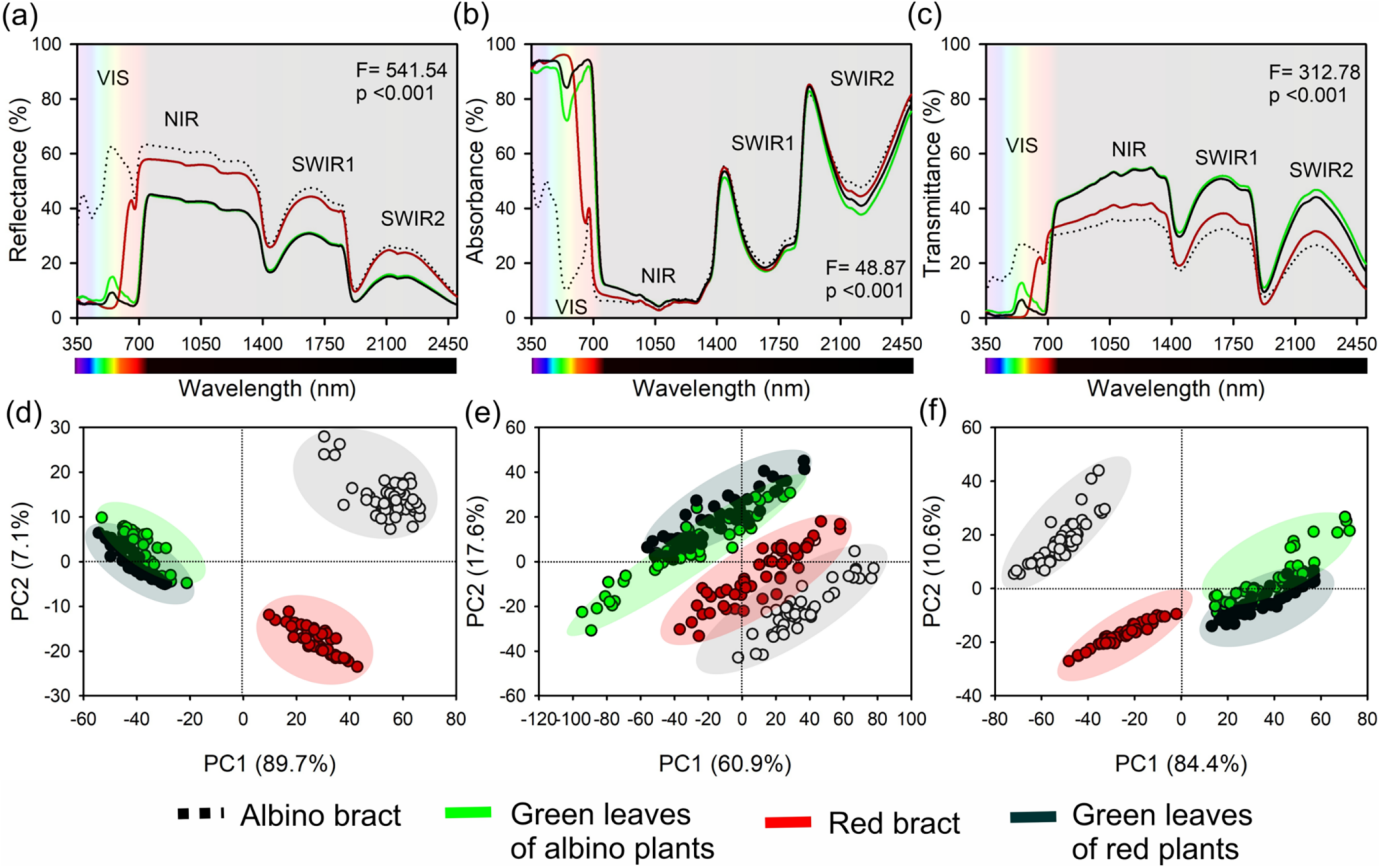
UV–VIS–NIR–SWIR hyperspectral (350–2500 nm) leaf optical traits and multivariate discrimination of poinsettia (*E. pulcherrima* Willd. ex Klotzsch) tissues. (a) Reflectance spectra. (b) Absorption spectra (Abs = 1 – (*R* + *T*), expressed as the fraction of incident radiation absorbed). (c) Transmittance spectra. Hyperspectral profiles of albino bracts, green leaves of albino plants, red bracts, and green leaves of red plants, with visible (VIS), near-infrared (NIR), and shortwave-infrared (SWIR1, SWIR2) regions indicated. (d) PCA of reflectance. (e) PCA of absorption. (f) PCA of transmittance. ANOVA detected strong tissue effects for reflectance, absorption, and transmittance, as determined by the *F* test (*p < 0.01*). Principal component analysis showing separation of the four tissue types along PC1 and PC2. *n* = 10 biological replicates per tissue for inferential statistics; the full set of spectra (*n* = 40 per tissue) was used for PCA visualization.

Principal component analysis of the full spectrum confirmed that optical traits alone were sufficient to discriminate the four tissue types (Figure 2d–f). For reflectance, PC1 (89.7%) and PC2 (7.1%) separated bracts from leaves and albino versus red phenotypes, respectively. PCA of absorption (PC1 = 60.9%, PC2 = 17.6%) highlighted the contrast between chlorophyll-rich leaves and chlorophyll-poor bracts, whereas for transmittance, PC1 (84.4%) described a gradient from highly transmitting AB to more opaque RB and PC2 (10.6%) further separated AG from RG. In all three PCAs, tissues formed nonoverlapping clusters (*n* = 40 spectra per tissue), demonstrating that pigment packaging and anatomical organization jointly generate distinct, statistically separable hyperspectral signatures (Figures 2a–f and S1) that anticipate the contrasting photosynthetic capacities quantified next via A–PAR and *A*–*C*_i_ analyses.

### 3.3 Photosynthetic Capacity and CO_2_ Assimilation

Gas exchange showed strong functional separation between bracts and green leaves (Figure 3a–c and Table S1). In both bract types, net CO_2_ assimilation remained near carbon balance and was slightly negative at high light (A1500 = −0.06 in AB and −0.09 in RB), consistent with very low *g*_s_ and *g*_m_ and minimal biochemical capacity (*V*_cmax_ ≈ 5; *J*_max_ ≈ 11–13; TPU < 1). Green leaves maintained positive assimilation across irradiances, with AG showing intermediate A1500 (5.10) and RG reaching the highest A1500 (15.57). This was matched by stepwise increases in *g*_s_/*g*_m_ and in Vcmax–Jmax–TPU (RG: *V*_cmax_ 68.37; *J*_max_ 127.59; TPU 14.17). Together, the A–PAR and A–C_i_ responses indicate that bracts are strongly sink- and diffusion-limited, whereas green leaves—especially RG—support high CO_2_ supply and carboxylative throughput.

**Figure 3:**
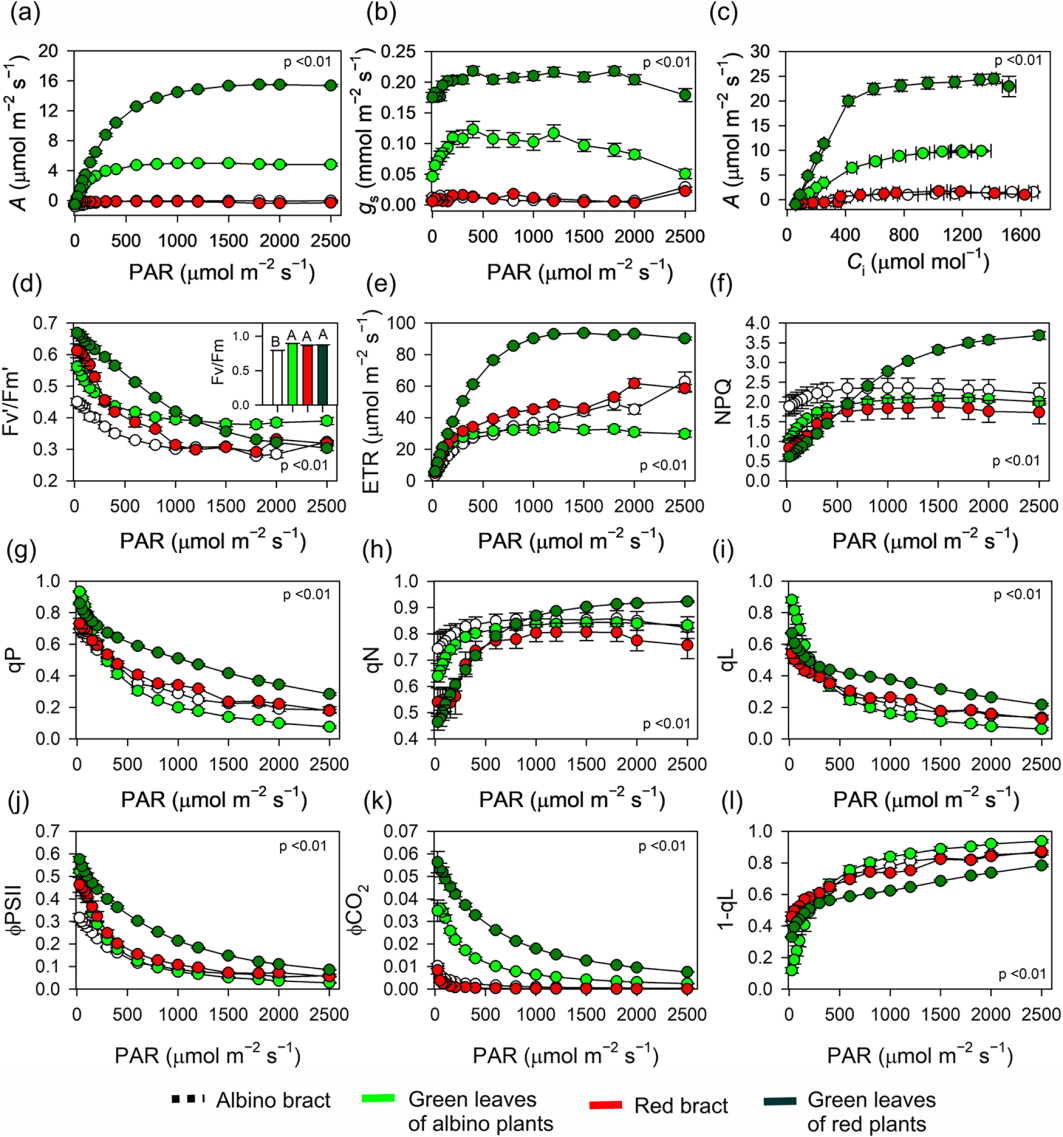
Photosynthetic, gas-exchange, and chlorophyll a fluorescence-derived photochemical and nonphotochemical responses to increasing PAR in contrasting poinsettia (*E. pulcherrima* Willd. ex Klotzsch) tissues. (a) Light-response curves of the net CO_2_ assimilation rate (*A*) as a function of photosynthetically active radiation (PAR; μmol m^–2^ s^–1^). (b) Light-response curves of stomatal conductance to water vapor (*g*_s_ mol m^–2^ s^–1^). (c) *A*–*C*_i_ curves of *A* versus the intercellular CO_2_ concentration (*C*_i_; μmol mol^–1^). (d) Effective quantum yield of PSII in the light (*F*_v_′/*F*_m_′); inset, dark-adapted maximum PSII efficiency (*F*_v_/*F*_m_). (e) Electron-transport rate (ETR). (f) Nonphotochemical quenching (NPQ). (g) Photochemical quenching coefficient (qP). (h) Nonphotochemical quenching coefficient (qN). (i) Fraction of open PSII centers (qL). (j) Quantum yield of PSII photochemistry (ΦPSII). (k) Apparent quantum yield of CO_2_ assimilation (ΦCO_2_). (l) 1–qL, estimating the reduction state of the PSII acceptor side. Curves are shown for albino bracts, green leaves of albino plants, red bracts, and green leaves of red plants (see the legend) to assess how pigment depletion and anthocyanin accumulation constrain light use, stomatal opening, and CO_2_ fixation. Symbols represent plant means ± SE (*n* = 10); each plant mean was derived from four organs per plant. Curves are shown for the albino bract, green leaves of albino plants, red bract, and green leaves of red plants, illustrating how pigment composition alters the balance between photochemistry and energy dissipation under increasing irradiance. Light-response curves differ among tissues, as determined by the Tukey test (*p* < 0.01).

Expressing the yield on an absorbed photon basis clarified this contrast. Green leaves combined high absorption with high CO_2_ return per absorbed photon (high αCO_2_,abs), whereas bracts either absorbed little (AB) or absorbed strongly via screening pigments (RB) yet converted virtually none of that absorbed energy into net CO_2_ gain. Consistent with these limitations, the intercellular CO_2_ required to approach saturation (*C*_iSAT_) was highest in albino bracts (≈1264 μmol mol^–1^) and lower in green leaves (≈657–804 μmol mol^–1^). Energy requirements for CO_2_ assimilation derived at *C*_iSAT_ likewise peaked in the green leaves of red plants (ATP_CO_2__ and NADPH_CO_2__) and were minimal in both bract types (Table S1). Accordingly, differences among tissues were driven more by CO_2_ diffusion and biochemical sink strength than by absorption or ΦPSII alone. This decoupling sets the stage for interpreting optical signatures in functional terms: hyperspectral profiles capture how light is absorbed and redistributed within tissues, but gas exchange is required to reveal whether that absorbed energy is channeled into carboxylation or diverted to dissipation and alternative sinks.

### 3.4 PSII Energy Partitioning and OJIP Transients

Chlorophyll fluorescence revealed similar dark-adapted PSII efficiency across tissues (*F*_v_/*F*_m_ ≈ 0.85; minimum 0.79 and maximum 0.89), but clear divergence under actinic light (Figure 3d–l and Table S1). Under high PAR, RG displayed the highest ΦPSII and ETR and also the highest ΦCO_2_, whereas both bract types retained measurable photochemistry but showed near-zero ΦCO_2_. Energy partitioning supported greater regulation in bracts: NPQ increased with irradiance in all tissues but remained lower in RB than in RG, and the excitation pressure (1 – qL) was comparatively high in tissues with low CO_2_ use, consistent with sink limitation.

Fast OJIP induction kinetics displayed the characteristic O–J–I–P rise in all tissues, but both bract types showed a steeper early rise and reduced relative fluorescence in the I–P region compared with the two green-leaf types (Figure S4a). In the JIP-test summary, green leaves—particularly green leaves of red plants—showed higher electron-transport-related descriptors (including ψ­(EO), φ­(EO), δ*R*_0_, and ρ*R*_0_), whereas bracts showed comparatively higher dissipation-related terms (Figure S4b). Thermal imaging further revealed distinct temperature patterns between bract and leaf regions (Figure S4c), and phenomenological energy-flux (“pipeline”) diagrams indicated larger trapped and transported fluxes (TR/CS and ET/CS) in the green leaves of red plants and proportionally larger dissipative fluxes (DI/CS) in the bracts (Figure S4d).

### 3.5 Leaf Surface, Mesophyll Architecture, and Chloroplast Ultrastructure

Epidermal micromorphology (including stomatal and trichome traits) differed among tissues (Figures S2 and S3). Leaf thickness and mesophyll organization are shown in Figure S1.

TEM revealed a steep gradient in chloroplast development across tissues (Figures 4 and S5). Bracts often lacked chloroplasts or contained small plastids with poorly developed thylakoids and abundant plastoglobuli, and electron-dense vacuolar inclusions were frequent in RB. In contrast, mesophyll cells in the two green-leaf types contained multiple lens-shaped chloroplasts aligned along the cell periphery with extensive grana and stromal lamellae and occasional starch grains. Chloroplast–mitochondrion–peroxisome associations were observed, particularly in green leaves, consistent with tighter coupling between photosynthesis and downstream metabolism.

**Figure 4:**
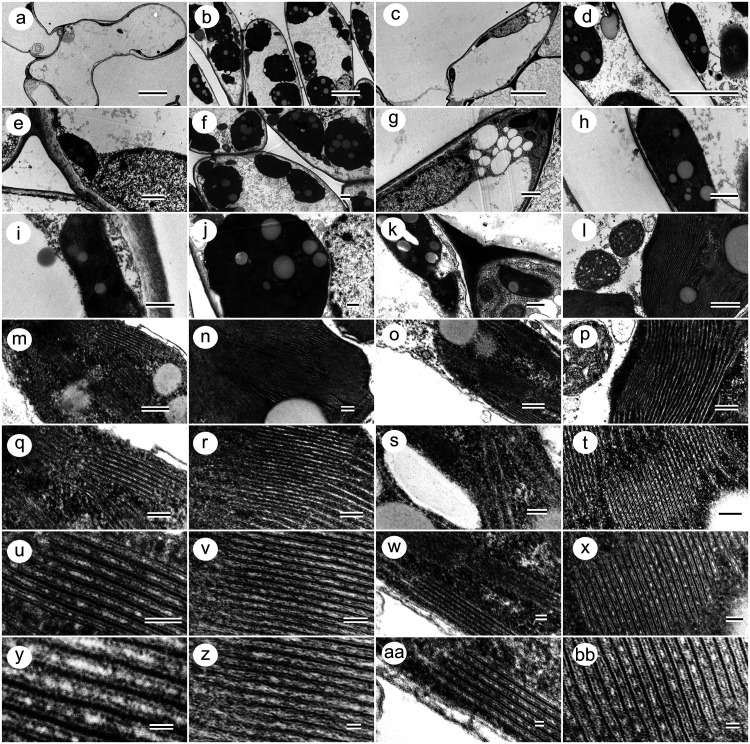
Transmission electron microscopy of the chloroplast ultrastructure in poinsettia (*E. pulcherrima* Willd. ex Klotzsch) leaves. The panels follow the same left-to-right order in all rows: albino bract (a, e, i, m, q, u, y), green leaves of albino plants (b, f, j, n, r, v, z), red bract (c, g, k, o, s, w, aa), and green leaves of red plants (d, h, l, p, t, x, bb). (a–d) Mesophyll cells showing the position of chloroplasts within the cell. (e–h) Whole chloroplasts, highlighting the envelope, stroma, plastoglobuli, and starch grains. (i–l) Higher magnification images of grana and stroma lamellae. (m–p) Granum stacks within the thylakoid system. (q–t) End-on views of grana showing lamella number and spacing. (u–x, y–bb) Increased magnification of the thylakoid membranes and lumen, revealing differences in grana compactness and membrane organization among tissues. Scale bars: 5 μm (a–d), 1 μm (e–h), 500 nm (i–l), 200 nm (m–p), 100 nm (q–t), 50 nm (u–x), and 20 nm (y–bb).

Ultrastructural quantification supported these patterns: green leaves had more chloroplasts per mesophyll cell and greater thylakoid/grana investment than either bract type, while thylakoid density varied less strongly among tissues (Figure S6). Together, these metrics indicate that the dominant difference among syndromes is the total thylakoid “hardware” deployed per cell and per lamina area, not simply local membrane packing. Surface traits (stomatal density, stomatal index, trichome density, and epidermal patterning) are summarized in Figure S6a–h.

### 3.6 Integrative PCA of Biochemical, Structural, and Physiological Traits

Principal component analysis integrating biochemical composition, structural and ultrastructural traits, gas exchange, and fluorescence explained 67.7% of the total variation on the first two axes (PC1, 42.4%; PC2, 25.3%) and clarified the coordinated trait syndromes underpinning variation in the photosynthetic quantum yield (αCO_2_,abs) (Figure 5a,b and Table S2). PC1 captured a gradient in pigment pools and chloroplast/thylakoid investment that covaried with CO_2_ diffusion and carboxylative capacity, and therefore aligned closely with αCO_2_,abs across tissues. PC2 was driven mainly by JIP-test descriptors and phenomenological energy-flux terms, reflecting reaction-center and cross-sectional excitation dynamics. In score space, albino and red bracts clustered at negative PC1 values, consistent with low pigment content, reduced chloroplast/grana development, and low conductance, with red bracts further displaced along PC2. Both green-leaf types separated at positive PC1 values, while green leaves of red plants tended toward higher PC2 scores. Overall, the ordination links αCO_2_,abs to coordinated pigment–structure syndromes that determine how efficiently absorbed energy is coupled to net CO_2_ fixation (Figure 5).

**Figure 5:**
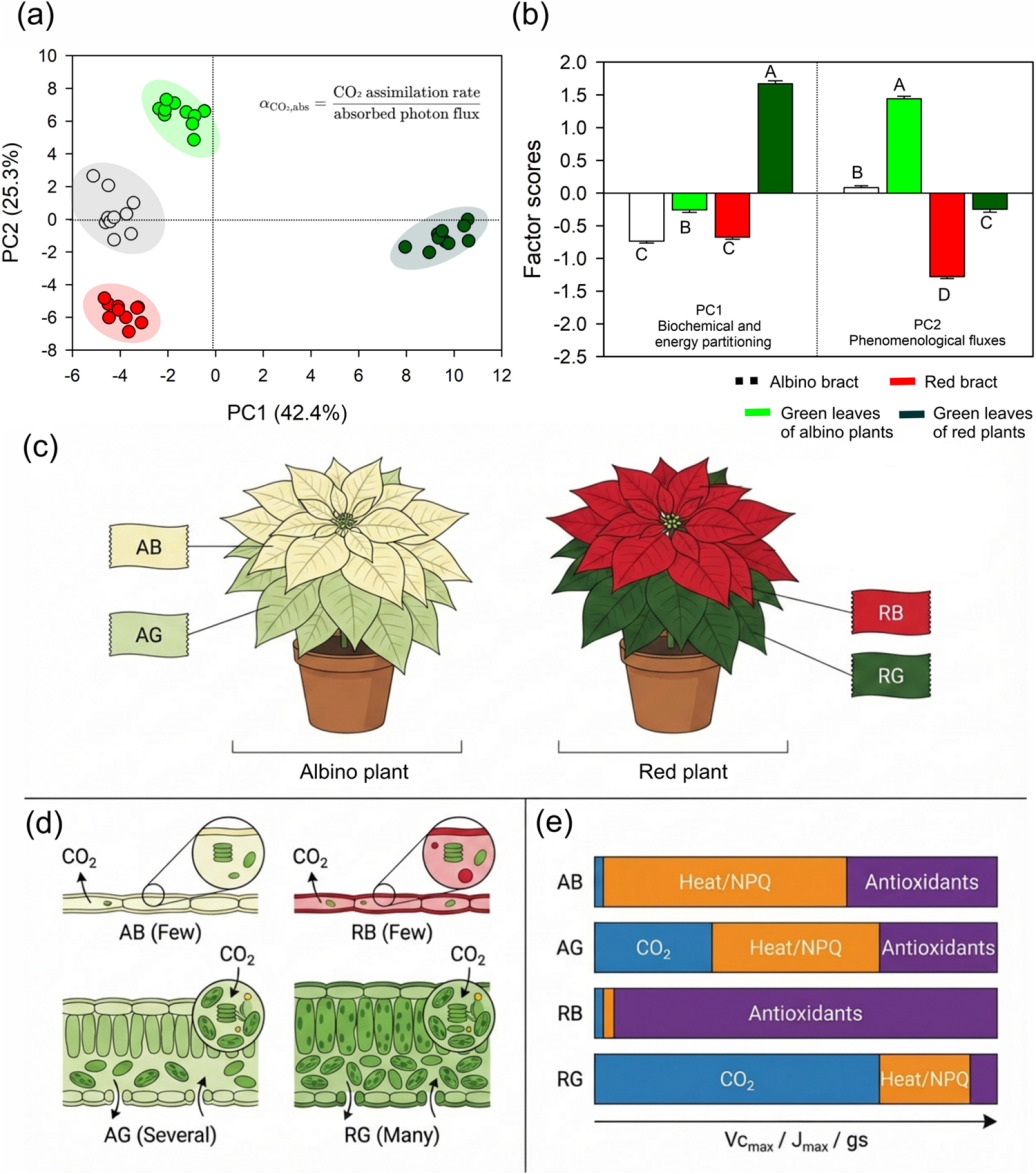
Multivariate integration of biochemical, structural, and photosynthetic traits in poinsettia (*E. pulcherrima* Willd. ex Klotzsch) leaves explaining the variation in photosynthetic quantum yield (αCO_2_,abs) and conceptual model linking pigment–structure syndromes to photosynthetic capacity and energy partitioning in poinsettia. (a) Principal component analysis (PCA) score plot for albino bracts, green leaves of albino plants, red bracts, and green leaves of red plants. Each point represents an individual leaf (*n* = 10); shaded ellipses indicate group dispersion. (b) Mean factor scores (means ± SE) for PC1 (“biochemical and energy partitioning”) and PC2 (“phenomenological fluxes”) for each tissue type. (c) Whole-plant phenotypes of an albino plant (left) and a red plant (right). Albino bracts (AB, cream) and green leaves of albino plants (AG, medium green) are contrasted with red bracts (RB, bright red) and green leaves of red plants (RG, dark green). (d) Idealized leaf cross sections illustrating tissue thickness, mesophyll organization, and chloroplast abundance. AB and RB have very thin laminae with few chloroplasts (“Few”), whereas AG has a thicker palisade–spongy mesophyll with several chloroplasts, and RG has the thickest mesophyll with many chloroplasts (“Many”). The arrows indicate CO_2_ flux from the atmosphere into the mesophyll. (e) For each tissue, horizontal stacked bars summarize the relative contribution of absorbed light to CO_2_ assimilation (blue), regulated heat dissipation via nonphotochemical quenching, NPQ (orange), and alternative antioxidant sinks (purple), scaled qualitatively to biochemical capacity (*V*_cmax_, *J*_max_, and *g*_s_). AB and RB behave as strong sinks with negligible CO_2_ fixation but high dissipation and antioxidant buffering, AG shows partial compensation with intermediate CO_2_ yield, and RG has the highest capacity and quantum yield for CO_2_ assimilation.

Different letters above the bars indicate significant differences among the tissues for each principal component (Tukey’s test, *p* < 0.01). Means ± SE (*n* = 10).

## 4 Discussion

Across the four tissues, we identify distinct pigment–structure syndromes in which pigment pools, mesophyll organization, and chloroplast ultrastructure covary to determine the absorbed-light quantum yield of CO_2_ assimilation (αCO_2_,abs) (Figures 1–5; and Table S1). Both bract types retain functional PSII but show low CO_2_ diffusion and minimal carboxylative capacity, so absorbed energy is preferentially dissipated or buffered by antioxidant sinks rather than converted into net carbon gain. Companion green leaves, particularly RG, increase the chloroplast/grana investment together with *g*_s_/*g*_m_ and *V*_cmax_–*J*_max_–TPU, partially compensating for the low-yield bracts at the module scale (Figure 5).

### 4.1 Pigment–Structure Syndromes and the Decoupling between Light Capture and Photosynthetic Yield

Pigment–structure syndromes reshape leaf radiative transfer by combining chlorophyll absorption, extra-chloroplastidic screening, and mesophyll scattering, yielding distinct UV–VIS–NIR–SWIR fingerprints (Figure 2).^15,41,42^

These optical fingerprints emerge because color is produced by both pigment abundance and pigment location: chlorophylls and carotenoids are packaged within thylakoids, while anthocyanins/phenolics are largely extra-chloroplastidic, often in epidermal or vacuolar compartments.^43,44^ In AB, reduced chlorophyll allows strong backscattering and transmission in the visible, whereas in RB, strong screening pigments can generate low reflectance and high visible absorption without a proportional increase in chloroplast investment.^8,17,19,45^ The NIR–SWIR domain adds complementary constraints linked to tissue thickness, airspace geometry, and water/structural absorptions, which helps discriminate tissues that may look similar in the visible but differ strongly in mesophyll organization. Together, this explains why the four tissue types form nonoverlapping clusters in spectral PCA space and why a “syndrome” framework (rather than single-wavelength indices) is more reliable for mixed colored canopies.

Yet optical phenotype and ΦPSII do not necessarily predict carbon return: in bracts, PSII remains photochemically competent, but low CO_2_ delivery and weak metabolic sink strength divert excitation away from net carboxylation (Figure 3 and Table S1).^10,46^

Albino and red bracts illustrate two routes to low photosynthetic yield: albino tissues minimize chlorophyll-based light harvesting, whereas red tissues impose strong anthocyanin/phenolic filtering, but both converge on sink-limited photochemistry and antioxidant buffering (Figure 1 and Table S1).^44,47,48^

Maintaining redox balance under sink limitation likely involves layered photoprotective processes (NPQ components and alternative electron routing) in addition to pigment screens, which helps explain why ΦPSII can remain measurable even when αCO_2_,abs collapses.^7,49,50^

A key implication is that ΦPSII and ETR report the behavior of the light reactions, whereas αCO_2_,abs integrates the full pathway from photon absorption to CO_2_ fixation. In sink-limited tissues, charge separation and electron flow can continue, while stromal demand for ATP/NADPH is low because CO_2_ delivery (*g*_s_ and *g*_m_), RuBP regeneration capacity (*J*_max_), and/or Rubisco capacity (*V*_cmax_) are constrained. Under these conditions, excess excitation must be managed to avoid overreduction, typically by increasing regulated thermal dissipation, engaging alternative electron pathways, and buffering reactive oxygen species with antioxidant systems. This helps reconcile the apparent paradox that bracts can show near-normal dark-adapted PSII efficiency yet contribute negligibly to the net CO_2_ gain under light.

### 4.2 Compensation by Green Companion Leaves and Whole-Plant Integration

Because bracts contribute little to net CO_2_ gain, whole-module carbon balance depends on companion green leaves acting as strong sources, consistent with broader evidence that nonfoliar tissues often rely on adjacent leaves for carbon.^2,10^

Compensation reflects the matched scaling of diffusive and biochemical limitations: RG increases *g*_s_ and *g*_m_ alongside *V*_cmax_–*J*_max_–TPU and sustains the highest ΦCO_2_ and iWUE (Figure 3 and Table S1).^10,51,52^ This coordination limits chronic overreduction while maintaining a regulated photoprotective buffer.^6,53,54^

The surface and anatomical traits reported here are consistent with this scaling. Bracts generally show a low stomatal occurrence and limited transpiration capacity, which constrains evaporative cooling and reinforces sink limitation under high irradiance. By contrast, green leaves combine higher stomatal conductance with a mesophyll structure that supports higher *g*_m_, allowing them to sustain CO_2_ delivery without a proportional penalty in water loss, as reflected by iWUE differences. Surface micromorphology (wax deposition, cell convexity, and trichomes) may further influence boundary-layer properties and leaf energy balance, helping explain why thermal patterns diverge between colored bracts and green foliage. Placing the detailed SEM and surface quantification in Figures S2, S4, and S6 keeps the main narrative focused on the coupling between diffusion, chloroplast investment, and αCO_2_,abs while retaining the structural evidence needed to interpret the gas-exchange and thermal signals.

Anatomy and ultrastructure provide the physical basis: thicker mesophyll and more grana-rich chloroplasts expand the chloroplast–airspace interface and support higher *g*_m_ and electron-transport capacity (Figures 4, S1, and S6).^51^ Such modular integration is expected in mixed canopies where productivity emerges from organ mosaics rather than a single leaf type.^55,56^

The compensation pattern also clarifies why canopy- or plant-level performance cannot be inferred from bracts alone. Because bracts are display tissues exposed to high irradiance, their stability requires protection against photooxidative stress, but their carbon balance is sustained by neighboring green leaves. In this context, RG appears to represent a high-throughput “source” state: higher conductance and biochemical capacity allow it to exploit absorbed light, while regulated dissipation remains available as a buffer. Some differences between AG and RG may reflect cultivar background, but the consistent bract-versus-leaf contrast within each phenotype supports a robust within-system inference: nongreen organs behave primarily as sinks, and green leaves adjust to meet the module’s carbon and redox demands.

This plant-strategy interpretation also depends on the developmental fate of the bracts. In poinsettia, the showy colored bracts are typically transient display leaves: after the flowering/display phase, bracts and leaves are progressively shed as the plant enters dormancy, whereas the next cycle begins with renewed vegetative green growth that only later produces new colored bracts under short-day induction.^10,46,49,50^ Thus, the small but functional chloroplast complement in AB and RB is best interpreted as protection of a temporary display organ through the reproductive/ornamental phase, rather than as investment in a long-lived leaf expected to recover full source function by direct regreening of the same bract.

### 4.3 Chloroplast Ultrastructure as a Determinant of Photosynthetic Quantum Yield

Across syndromes, chloroplast deployment and grana investment set a “hardware ceiling” for photosynthetic capacity, so αCO_2_,abs tracks chloroplast number and thylakoid investment more closely than absorption alone (Figures 4 and S6).^8^ Differences largely reflect total membrane built per cell rather than local packing density.^8,9,45^

Importantly, the “hardware ceiling” set by chloroplast number and grana development also provides a structural explanation for differences in fluorescence-derived parameters. When thylakoid investment is low, the capacity to distribute excitation and support downstream transport is reduced; therefore, even modest absorbed photon fluxes can elevate the excitation pressure and increase the need for regulation. Conversely, increasing thylakoid area and grana organization supports higher ETR and provides additional flexibility for balancing photochemistry with protective dissipation. In this framework, dark-adapted *F*_v_/*F*_m_ is expected to be relatively insensitive to chloroplast abundance because it reflects the efficiency of centers that are present, whereas light-adapted parameters (ΦPSII, qL, NPQ) respond strongly to the balance between excitation supply and stromal sink demand. This helps interpret why bracts can appear “photochemically competent” yet remain functionally nonassimilating.

Bracts can show near-normal *F*_v_/*F*_m_ because the PSII centers that form are competent, yet low abundance plus restricted CO_2_ delivery keep stromal sinks weak, forcing dissipation/alternative sinks under light (Figure 3 and Table S1).^10^ Limited appressed membranes constrain photosynthetic supercomplex connectivity and downstream transport.^6,45,57,58^ Reduced chloroplast coverage at the mesophyll surface further limits effective CO_2_ diffusion to chloroplasts.^43,51^

By contrast, green leaves tile multiple chloroplasts along the cell periphery, increasing the chloroplast–airspace interface and mesophyll conductance.^10,51,59^ They also invest more thylakoid area to sustain linear electron transport and ATP/NADPH supply matched to carbon metabolism.^6,60,61^ Membrane organization and redox routing then support both high ΦPSII/ΦCO_2_ and regulated photoprotection under high light.^45,62,63^ This is consistent with strong photosynthetic performance that co-occurs with flexible regulation.^35,64^ Overall, these patterns match broader links between structure, diffusion, and photosynthetic return across canopies.^10,51,59,65,66^

The ultrastructural data provide a mechanistic bridge between the anatomy and gas exchange. Increasing chloroplast number per mesophyll cell and increasing grana development expand the effective thylakoid area available for electron transport, raising the upper bound on the ATP/NADPH supply. At the same time, positioning multiple chloroplasts along the cell periphery increases chloroplast exposure to intercellular airspaces, which supports a higher effective mesophyll conductance and stronger coupling between diffusion and biochemistry. In our integrative PCA, these structural and ultrastructural metrics covaried with Vcmax–Jmax–TPU and αCO_2_,abs, reinforcing that photosynthetic yield in this system is determined less by color or absorption alone and more by the coordinated buildup of diffusion capacity and chloroplast “hardware”.

### 4.4 Anthocyanin-Rich Red Bracts: Photoprotective, Antioxidant Sinks Rather Than Efficient Photosynthetic Organs

Red bracts combine low chloroplast investment with strong vacuolar anthocyanin/phenolic pools, prioritizing optical filtering and redox buffering over carbon gain.^1,11,44,47^ Anthocyanins likely act as both screens and antioxidant reservoirs in tissues with intrinsically weak CO_2_ sinks.^44,47^ This buffering can stabilize photosystems under sustained illumination when excess excitation must be dissipated or routed to alternative sinks.^11,44,47,48^

Functionally, red bracts illustrate sink-limited photochemistry: electron transport remains measurable, while ΦCO_2_ is ∼0 because thin, chloroplast-poor mesophyll limits both membrane “hardware” and stromal demand.^67–69^ Regulation therefore depends on NPQ and alternative electron pathways that maintain ΔpH and redox balance.^7,49,50^ For remote sensing, this helps explain why strong visible absorption can overpredict photosynthetic capacity unless models account for pigment–structure syndromes.^15,18,70,71^

Anthocyanin-rich bracts therefore appear best interpreted as photoprotective, antioxidant sinks. Screening pigments reduce the photon load reaching the small chloroplast complement while also increasing the fraction of absorbed energy that must be dissipated or safely redirected. At the same time, high phenolic/anthocyanin pools provide a chemical buffering capacity for oxidative pressure, which may be particularly important during bract development when metabolism and transport are reprogrammed. This dual optical–chemical strategy can stabilize colored display organs under field-like irradiance, even though it yields little direct carbon gain. Recognizing this role helps avoid overinterpreting “strong absorption” as “high photosynthesis” in colored tissues.

### 4.5 Implications for Hyperspectral Monitoring and Colored Canopies

Across tissues, αCO_2_,abs reflects coordinated variation in pigment pools, structure/ultrastructure, CO_2_ diffusion, and energy partitioning, so it cannot be inferred from “greenness” or any single spectral metric.^41,45,52,72^ Accordingly, colored organs can decouple reflectance/absorption from carbon return, and hyperspectral interpretation should be syndrome-aware rather than assuming a universal reflectance–photosynthesis relationship.^14,15,56^ This is especially relevant for mixed pixels where strong visible absorption can come from vacuolar screening pigments without parallel increases in chloroplast investment or CO_2_-use capacity.^14,15,56^

Full-range spectra integrate pigment absorption and structural/water features, enabling classification (and potential unmixing) of colored organs versus assimilating foliage when coupled to physiological constraints.^6,14,16^

In practice, syndrome-aware monitoring can be implemented on multiple scales. At close range, hyperspectral imaging can map tissue types and quantify within-organ heterogeneity, while concurrent fluorescence (including rapid induction kinetics) provides constraints on PSII regulation and alternative sinks. At canopy scale, combining spectral classification with fluorescence- and temperature-based indicators can help separate shifts in pigment screening from shifts in carbon exchange, particularly during developmental transitions when colored organs expand. Because the key functional contrasts in this system are large (near-zero versus high CO_2_ uptake), even relatively simple models can benefit from first identifying whether a pixel is dominated by bracts or green leaves. More mechanistic retrievals can then focus on estimating *g*_s_/*g*_m_ and photosynthetic capacity within the green-leaf component while treating bract-dominated components as energy-dissipating sinks.

At canopy scale, combining hyperspectral classification with constraints on energy partitioning can reduce overestimation of productivity in anthocyanin-dominated scenes.^6,14,16^ For breeding and phenotyping, syndrome-based interpretation helps distinguish productive sources from photoprotective display sinks and their compensatory modules.^48,52,73^

For remote sensing and phenotyping, a practical workflow is to first identify the tissue mixture (e.g., green leaves versus colored bracts) using full-range spectra or multivariate classifiers and then apply syndrome-specific links to function. In poinsettia, the key functional axis aligns with CO_2_ diffusion (*g*_s_/*g*_m_), biochemical capacity (*V*_cmax_–*J*_max_–TPU), and chloroplast/thylakoid investment, whereas fluorescence and thermal signals provide complementary constraints on energy partitioning and dissipation. This two-step approach should reduce bias when colored organs dominate the field of view and should improve transferability across genotypes and developmental stages because it explicitly separates “optical appearance” from “carbon return”.

In summary, bracts retain PSII activity but αCO_2_,abs is set primarily by chloroplast abundance, thylakoid/grana investment, and CO_2_ conductance. Albino and red bracts behave as photoprotective, antioxidant sinks with near-zero net CO_2_ assimilation, while companion green leaves—especially RG—compensate via higher *g*_s_/*g*_m_ and *V*_cmax_–*J*_max_–TPU. These syndromes leave distinct hyperspectral fingerprints to guide interpretation of canopies containing colored or chlorophyll-deficient organs. This interpretation is consistent with a transient display organ strategy in which existing bracts are ultimately shed and replaced by new green vegetative growth in the next cycle.^10,46,49,50^

## Supplementary Material



## Data Availability

The data supporting the findings of this study are available within the paper and its Supporting Information.

## Notes

Leaf cross-sectional anatomy and thickness in poinsettia (*Euphorbia pulcherrima* Willd. ex Klotzsch) tissues; scanning electron microscopy of epidermal surfaces in poinsettia; chlorophyll a fluorescence kinetics and JIP-test energy-flux parameters in contrasting poinsettia tissues; photochemical, carboxylative, and PSII energy-partitioning parameters derived from A–PAR, *A*–*C*_i_, and chlorophyll a fluorescence in albino bracts, green leaves of albino plants, red bracts, and green leaves of red plant (*Euphorbia pulcherrima* Willd. ex Klotzsch) leaves; and relative contribution of functional trait groups to the first two principal components (PC1 and PC2) from the PCA integrating leaf traits underpinning the photosynthetic quantum yield (PDF)
