# Supplementary material for: Pigment–Structure Leaf Syndromes in Poinsettia (Euphorbia pulcherrima Willd. ex Klotzsch) Alter Chloroplast Ultrastructure, Biochemical Profiling, and Photosynthetic Quantum Yields
Source: ACS Omega. 2026 May 4;11(19):28909–22. doi: 10.1021/acsomega.6c01933 (PMC13191521; doi:10.1021/acsomega.6c01933)
Supplement: Supplementary file 1 [file ao6c01933_si_001.pdf]

Supplementary files

Running title: Pigment–structure leaf syndromes and photosynthetic yield dynamics

Title: Pigment–structure leaf syndromes in Poinsettia (*Euphorbia pulcherrima* Willd. ex Klotzsch) alter chloroplast ultrastructure, biochemical profiling and photosynthetic quantum yields

Renan Falcioni[1,2*], Werner Camargos Antunes[1,2], José Alexandre M. Demattê[3] and Marcos Rafael Nanni[1]

[1] Graduate Program in Agronomy, State University of Maringá, Av. Colombo, 5790, Maringá 87020−900, Paraná, Brazil

[2] Department of Biology, State University of Maringá, Av. Colombo, 5790, Maringá 87020−900, Paraná, Brazil

[3] Department of Soil Science, Luiz de Queiroz College of Agriculture, University of São Paulo, Av. Pádua Dias, 11, Piracicaba 13418−260, São Paulo, Brazil

[*]Correspondence:
Renan Falcioni; renanfalcioni@gmail.com or rfalcioni2@uem.br
Phone: +55 44 3011−1359
https://orcid.org/0000-0002-2343-5045

Supplementary Figures

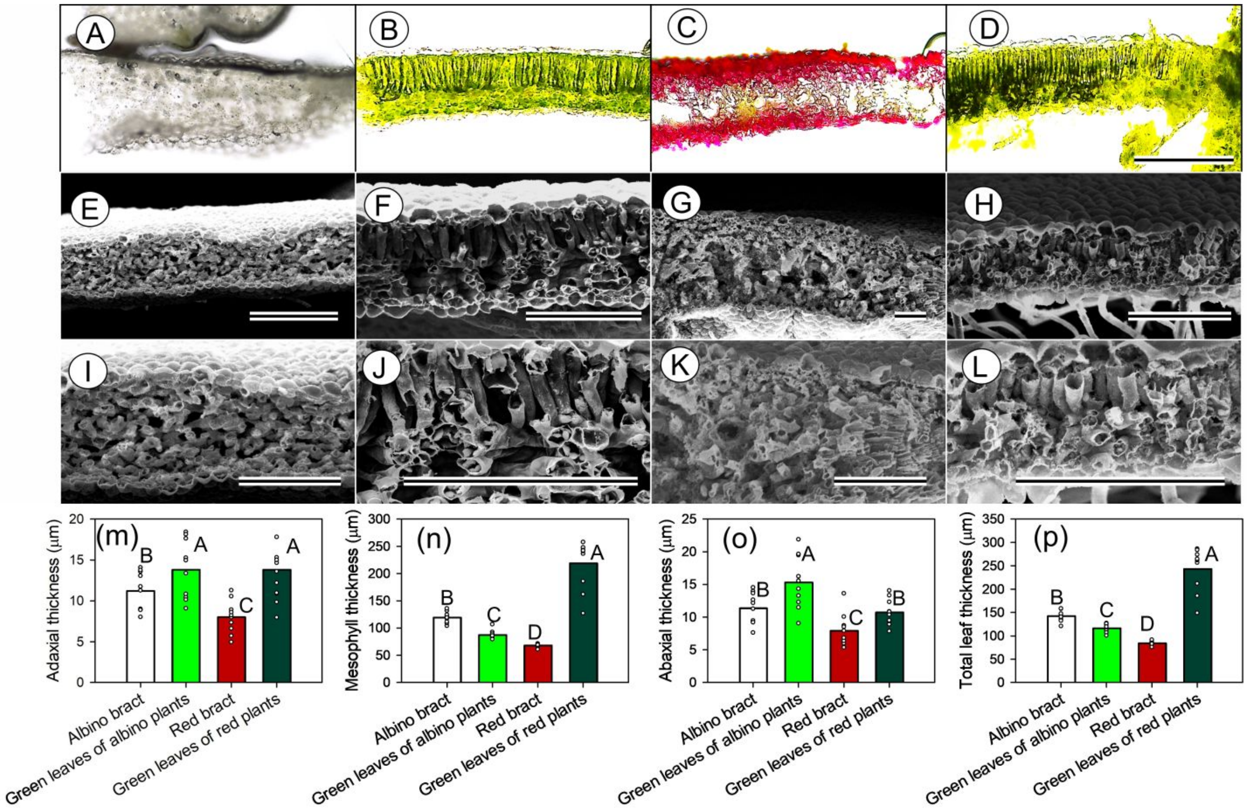

Figure S1 Leaf cross-sectional anatomy and thickness in poinsettia (*Euphorbia pulcherrima* Willd. ex Klotzsch) tissues. The panels follow the same left-to-right order: albino bract, green leaves of albino plants, red bract and green leaves of red plants. **(a–d)** Bright-field transverse sections. **(e–h)** SEM cross-sections at lower magnification. **(i–l)** SEM cross-sections at higher magnification, highlighting the adaxial and abaxial epidermis and mesophyll organisation used to relate the pigment phenotype to light path length and CO_2 diffusion. **(m–p)** Thickness of the adaxial epidermis, mesophyll, abaxial epidermis and total leaf, respectively. Bars are means with individual values. Means ± SE (n = 10), and different letters indicate significant differences among tissues (Tukey's test, $p < 0.01$). Scale bars: 150 µm for all micrographs.

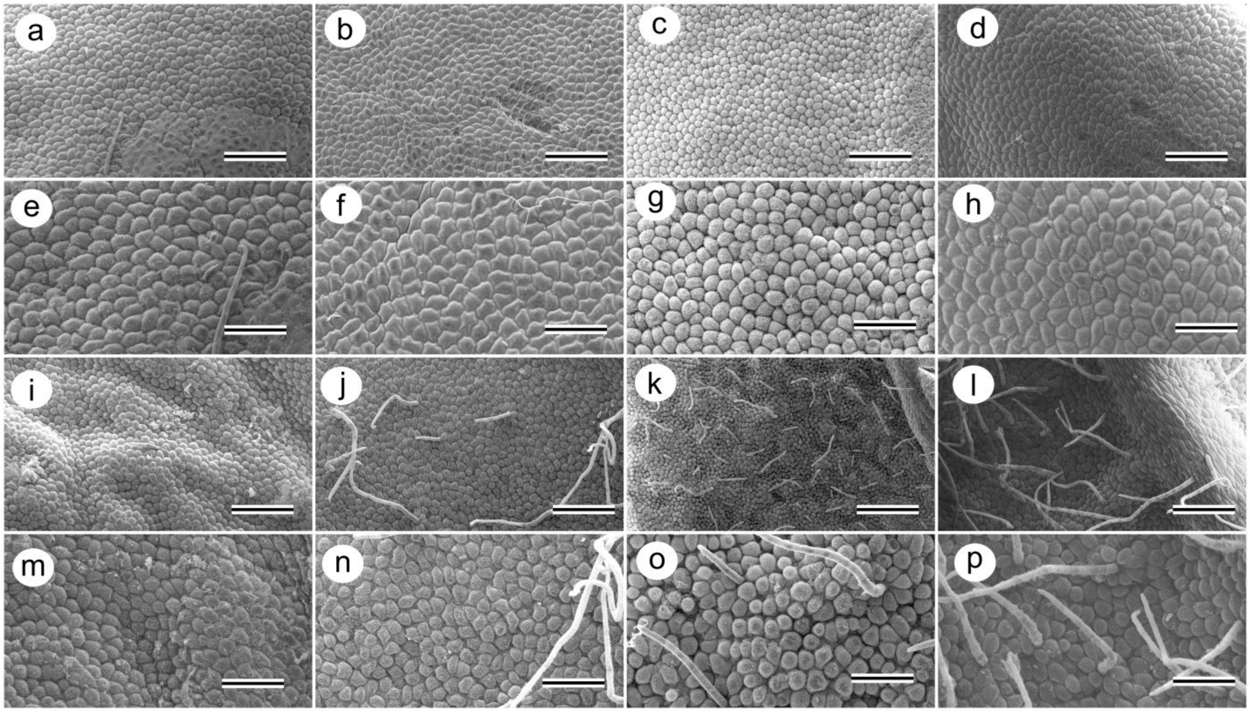

Figure S2 Scanning electron microscopy of epidermal surfaces in poinsettia (*Euphorbia pulcherrima* Willd. ex Klotzsch) leaves. Panels follow the same left-to-right order in all rows: albino bract **(a, e, i, m)**, green leaves of albino plants **(b, f, j, n)**, red bract **(c, g, k, o)** and green leaves of red plants **(d, h, l, p)**. **(a–h)** Adaxial surface; **(i–p)** abaxial surface. The first and third rows show lower magnification, and the second and fourth rows show higher magnification, highlighting the epidermal cell shape and trichome distribution. Scale bars: 200 μm **(a–d, i–l)** and 100 μm **(e–h, m–p)**.

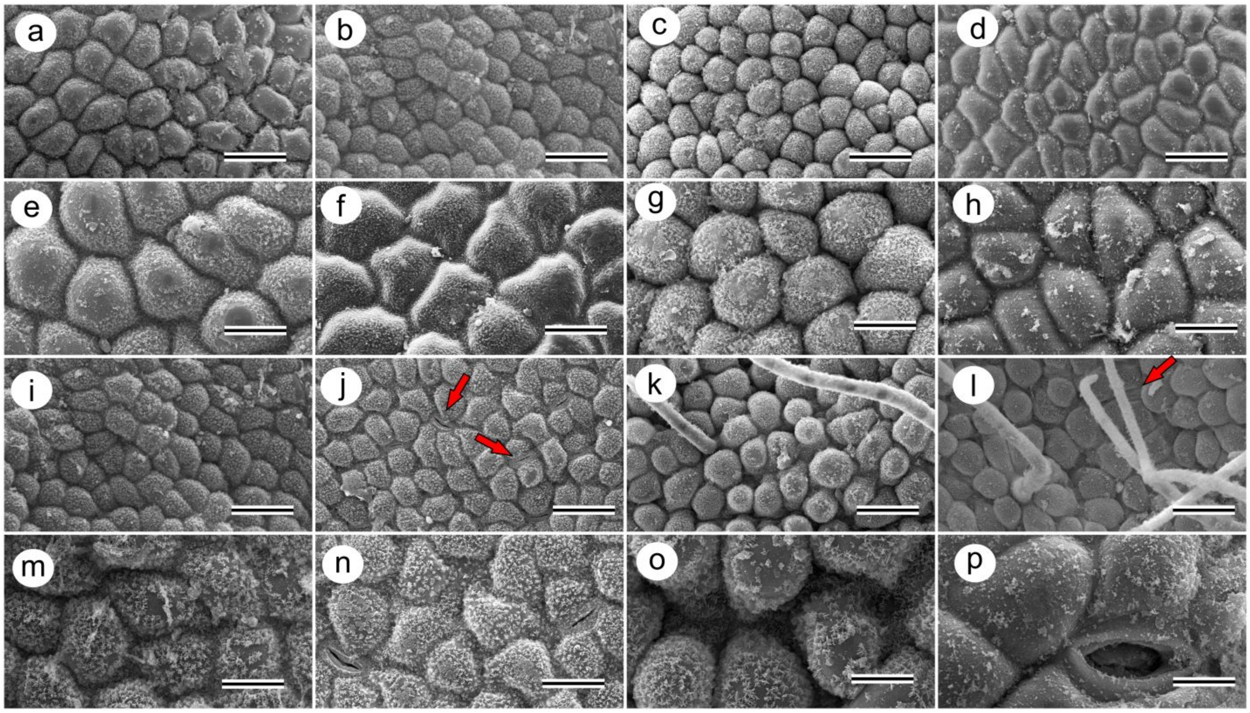

Figure S3 High-magnification scanning electron microscopy of epidermal surfaces in poinsettia (*Euphorbia pulcherrima* Willd. ex Klotzsch) leaves. The panels follow the same left-to-right order: albino bract, green leaves of albino plants, red bract and green leaves of red plants. **(a–h)** Adaxial surface; **(i–p)** abaxial surface. The first and third rows **(a–d, i–l)** show lower magnification, and the second and fourth rows **(e–h, m–p)** show higher magnification, indicating epidermal cell convexity, cuticular ornamentation and epicuticular wax deposition. The red arrows in **(j)** and **(l)** indicate abaxial stomata and associated wax accumulation, which are used to relate surface structure to gas exchange capacity. Scale bars: 50 μm **(a–d, i–l)** and 25 μm **(e–h, m–p)**.

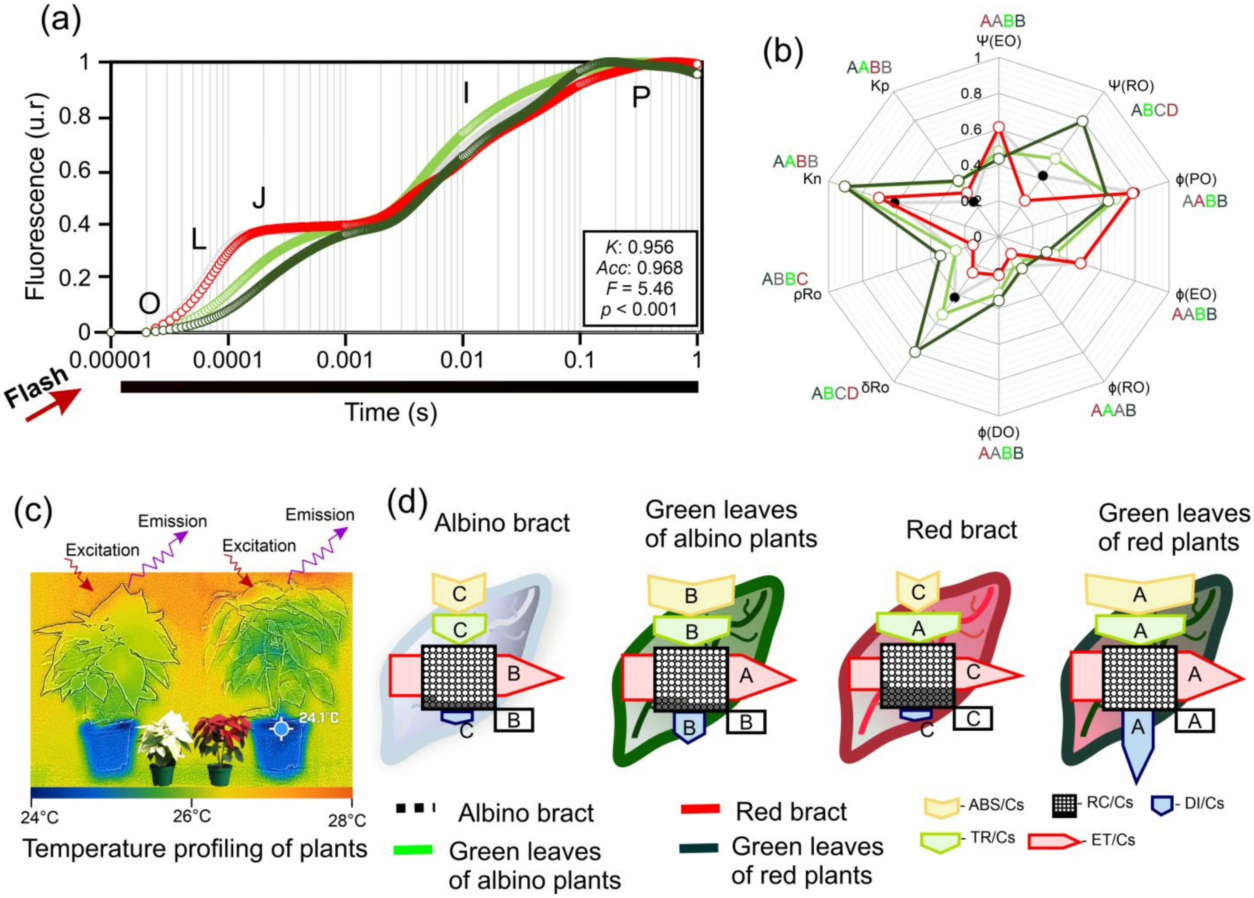

Figure S4 Chlorophyll *a* fluorescence kinetics and JIP-test energy-flux parameters in contrasting poinsettia (*Euphorbia pulcherrima* Willd. ex Klotzsch) tissues. **(a)** Chlorophyll *a* fluorescence induction kinetics (OJIP transients; relative fluorescence, log time scale) for albino bracts, green leaves of albino plants, red bracts and green leaves of red plants. **(b)** Radar plot of selected JIP-test parameters [ψ(EO), φ(EO), φ(PO), φ(RO), δR0, ρR0, Kp, Kn], indicating changes in PSII excitation energy trapping, electron transport and recombination among tissues; different letters on each axis denote significant differences. **(c)** Quantitative false-colour thermal images of albino and red plants acquired with an infrared thermal camera (FLIR), showing canopy temperature profiles under illumination and illustrating how differences in photon capture by chlorophylls and anthocyanins in bracts and green foliage translate into altered heat dissipation. The dashed line delineates the regions of albino bracts within green leaves and the regions of red bracts within green leaves, in both plants, respectively. **(d)** Pipeline–leaf diagrams showing phenomenological energy flows per excited PSII cross-section (CS): yellow arrow, absorbed energy (ABS/CS); green arrow, trapped energy leading to QA reduction (TR/CS); red arrow, electron transport beyond QA^-

(ET/CS); blue arrow, dissipated energy (DI/CS); squares with circles, fraction of QA-reducing reaction centres (RC/CS). Arrow size and RC symbols indicate the redistribution of absorbed energy between photochemistry and non-photochemical dissipation among tissues, as determined by Tukey's test ($p < 0.01$). Means ± SE (n = 10).

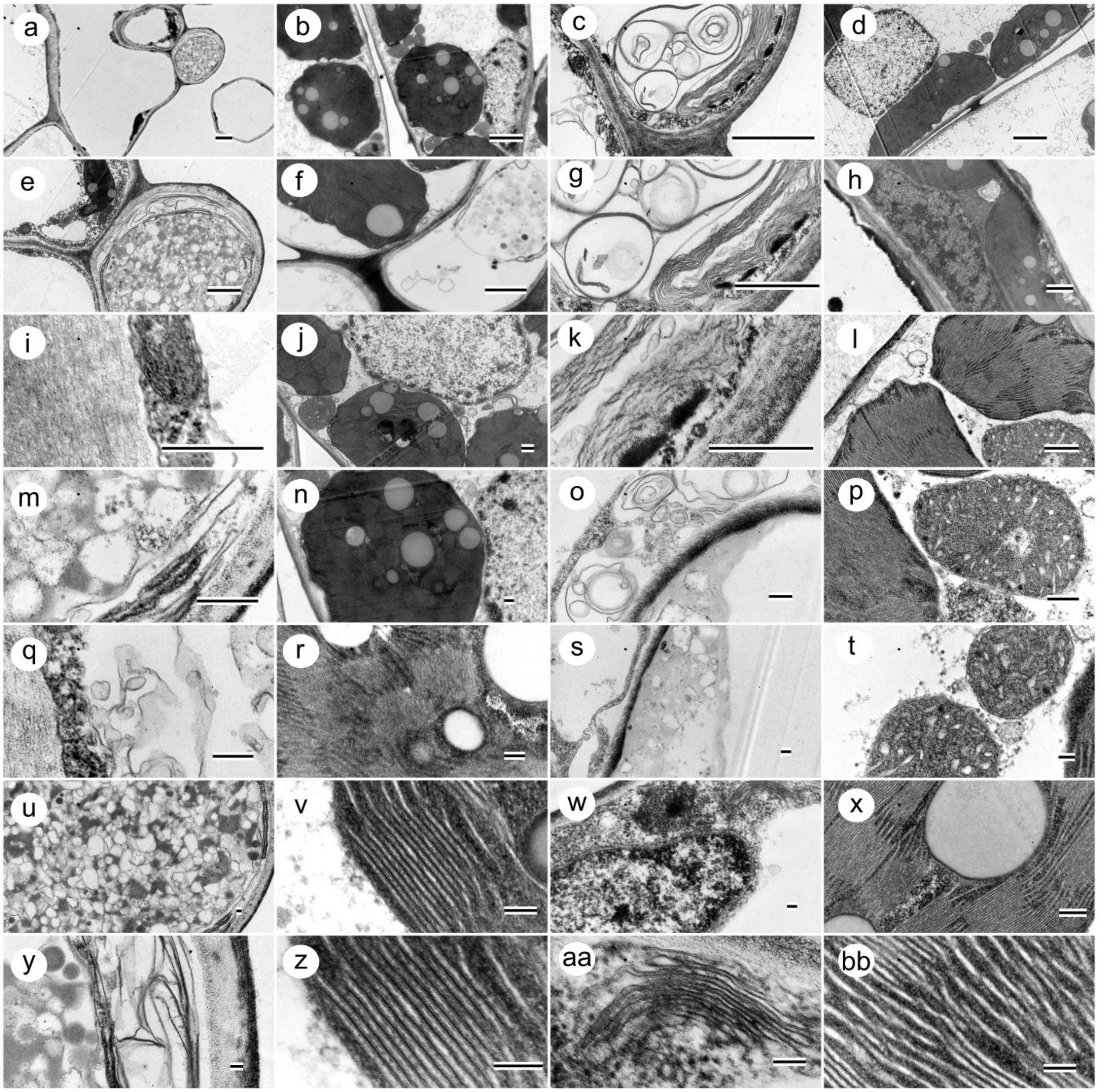

Figure S5 Transmission electron microscopy of chloroplasts and associated organelles in mesophyll cells of poinsettia (*Euphorbia pulcherrima* Willd. ex Klotzsch) leaves. The panels follow the same left-to-right order in all rows: albino bract **(a, e, i, m, q, u, y)**, green leaves of albino plants **(b, f, j, n, r, v, z)**, red bract **(c, g, k, o, s, w, aa)** and green leaves of red plants **(d, h, l, p, t, x, bb)**. **(a–d)** Mesophyll regions showing the spatial associations of chloroplasts with mitochondria, peroxisomes and vacuoles. **(e–h)** Individual chloroplasts adjacent to mitochondria/peroxisomes and large vacuoles, including phenolic- or anthocyanin-containing vacuoles in red tissues. **(i–l)** Higher-magnification images of chloroplast envelopes and

neighbouring organelles. **(m–p)** Chloroplast–mitochondrion–peroxisome complexes involved in photorespiratory metabolism. **(q–t)** Details of organelle membranes and contact sites. **(u–bb)** Increased magnification of thylakoid systems, plastoglobuli and mitochondrial cristae, highlighting shifts in grana organisation, lipid bodies and organelle coupling between pigment-deficient and anthocyanin-rich tissues. Scale bars: 2 μm **(a–d)**, 1 μm **(e–h)**, 500 nm **(i–l)**, 300 nm **(m–p)**, 200 nm **(q–t)** and 100 nm **(u–bb)**.

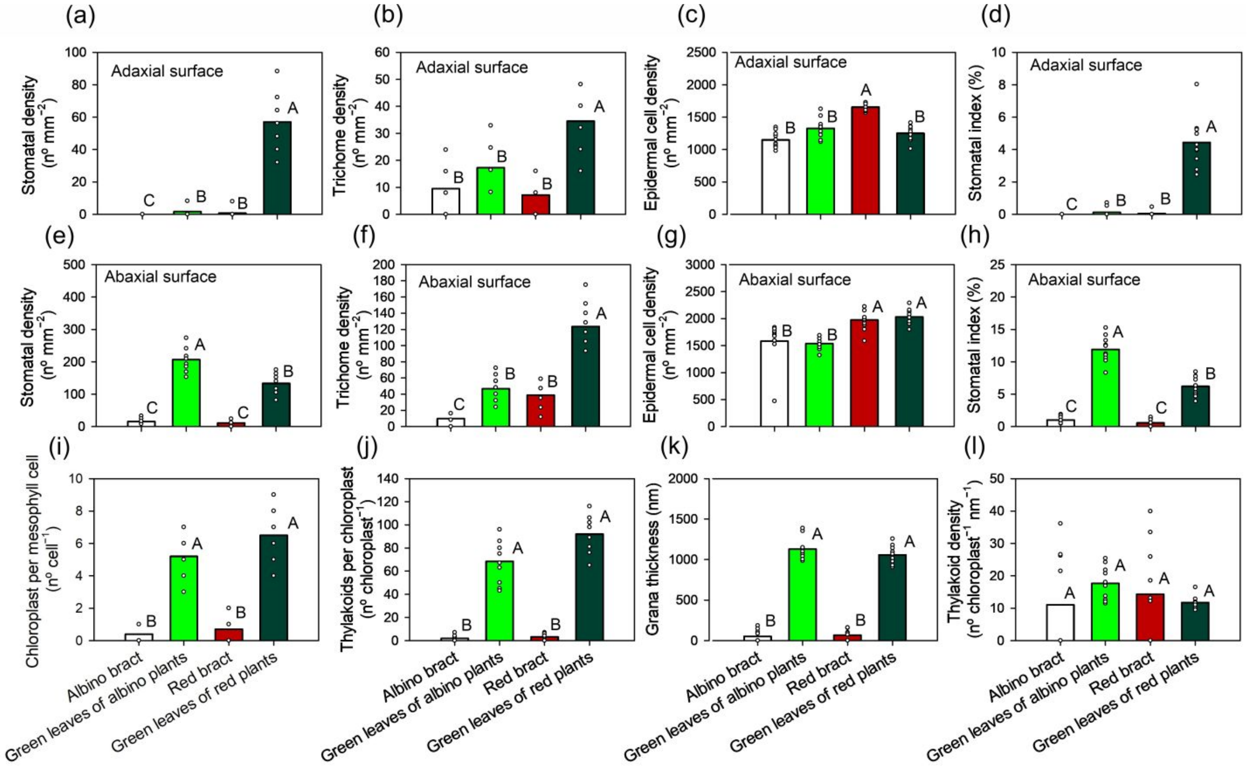

Figure S6 Surface and chloroplast structural traits in poinsettia (*Euphorbia pulcherrima* Willd. ex Klotzsch) leaves. **(a–d)** Adaxial surface. **(a)** Stomatal density (n° mm^{-2}). **(b)** Trichome density (n° mm^{-2}). **(c)** Epidermal cell density (n° mm^{-2}). **(d)** Stomatal index (%). **(e–h)** Abaxial surface. **(e)** Stomatal density (n° mm^{-2}). **(f)** Trichome density (n° mm^{-2}). **(g)** Epidermal cell density (n° mm^{-2}). **(h)** Stomatal index **(%)**. **(i–l)** Mesophyll chloroplast architecture. **(i)** Chloroplasts per mesophyll cell (n° $cell^{-1}$). **(j)** Thylakoids per chloroplast (n° $chloroplast^{-1}$). **(k)** Grana thickness (nm). **(l)** Thylakoid density (n° $chloroplast^{-1}$ nm^{-1}). The bars represent the means ± SE, and the circles represent individual measurements of albino bracts, green leaves of albino plants, red bracts and green leaves of red plants. Different uppercase letters indicate significant differences among the tissues within each panel (Tukey's test, $p < 0.01$). Means ± SE (n = 10).

Supplementary Tables

Table S1 Photochemical, carboxylative and PSII energy-partitioning parameters derived from A–PAR, A–Ci and chlorophyll a fluorescence in albino bracts, green leaves of albino plants, red bracts and green leaves of red plants (Euphorbia pulcherrima Willd. ex Klotzsch) leaves.

**Parameters**		**Albino bract**				**Green leaves of albino plants**				**Red bract**				**Green leaves of red plants**			
**Photochemical variables (*A*-PAR)**	Rd	0.78	±	0.02	A	0.45	±	0.07	B	0.29	±	0.01	C	0.42	±	0.04	B
	LCP	338.61	±	55.64	A	12.91	±	2.16	C	234.43	±	33.21	B	10.51	±	1.08	C
	LSP	1960.67	±	13.83	A	402.11	±	43.93	C	1934.51	±	8.22	A	613.74	±	24.01	B
	PN_{MAX}	5.90	±	1.17	B	5.61	±	0.20	B	3.16	±	0.32	C	16.96	±	0.19	A
	A_{MAX}	-0.06	±	0.05	C	5.10	±	0.15	B	-0.09	±	0.01	D	15.57	±	0.15	A
	α	0.003	±	0.000	C	0.035	±	0.001	B	0.001	±	0.000	D	0.041	±	0.002	A
	ATP	27.28	±	2.68	B	12.86	±	0.70	C	24.02	±	1.03	B	35.45	±	0.21	A
	NADPH	18.19	±	1.79	B	8.58	±	0.47	D	16.01	±	0.69	C	23.63	±	0.14	A
	i*WUE*	-12.20	±	7.69	C	58.01	±	8.41	B	-61.79	±	18.04	D	74.36	±	2.11	A
**Carboxylative CO_2 assimilation (*A*-C_i)**	Rd^*_{CO2}	0.99	±	0.15	D	1.50	±	0.19	B	1.24	±	0.11	C	2.84	±	0.05	A
	VCMAX	5.15	±	1.27	C	25.33	±	2.58	B	5.36	±	0.49	C	68.37	±	2.62	A
	TPU	0.84	±	0.06	D	5.21	±	1.06	B	0.92	±	0.07	C	14.17	±	3.29	A

	JMAX	10.71	±	0.87	C	54.82	±	4.11	B	12.77	±	0.92	C	127.59	±	5.03	A
	g_s	0.005	±	0.001	D	0.065	±	0.008	B	0.007	±	0.002	C	0.152	±	0.010	A
	g_m	0.039	±	0.028	C	0.342	±	0.089	B	0.003	±	0.001	C	1.396	±	0.284	A
	Cc	0.68	±	0.08	A	0.34	±	0.05	C	0.59	±	0.05	B	0.18	±	0.01	D
	Γ	257.62	±	12.00	A	75.71	±	49.20	B	90.13	±	3.42	B	273.90	±	36.54	A
	C_{iSAT}	1263.55	±	46.67	A	656.58	±	1.26	C	833.95	±	90.35	B	803.74	±	0.23	B
	ATP_{CO2}	4.01	±	0.33	C	20.56	±	1.54	B	4.79	±	0.34	C	47.85	±	1.89	A
	$NADPH_{CO2}$	2.68	±	0.22	C	13.70	±	1.03	B	3.19	±	0.23	C	31.90	±	1.26	A
**Chlorophyll a fluorescence - PSII energy partitioning at 1500 µmol m^{-2} s^{-1}**	Fv'/Fm'	0.31	±	0.01	A	0.38	±	0.01	A	0.31	±	0.01	A	0.36	±	0.01	A
	ETR	44.00	±	2.40	B	32.20	±	2.14	C	45.80	±	1.29	B	93.68	±	0.66	A
	NPQ	2.34	±	0.25	B	2.10	±	0.08	C	1.88	±	0.28	D	3.32	±	0.07	A
	qP	0.225	±	0.014	B	0.139	±	0.013	C	0.236	±	0.005	B	0.416	±	0.007	A
	qN	0.85	±	0.03	A	0.84	±	0.01	A	0.81	±	0.03	A	0.90	±	0.00	A
	qL	0.170	±	0.010	B	0.111	±	0.013	C	0.177	±	0.004	B	0.315	±	0.007	A
	ΦPSII	0.070	±	0.004	B	0.051	±	0.003	C	0.072	±	0.002	B	0.148	±	0.001	A
	ΦCO_2	0.0006	±	0.0001	C	0.0043	±	0.0002	B	0.0001	±	0.0000	D	0.0126	±	0.0001	A
	1-qL	0.830	±	0.010	B	0.889	±	0.013	A	0.823	±	0.004	B	0.685	±	0.007	C

Table S2 Relative contribution of functional trait groups to the first two principal components (PC1 and PC2) from the PCA integrating leaf traits underpinning the photosynthetic quantum yield (αCO_2,abs = CO_2 assimilation rate/absorbed photon flux) in poinsettia (Euphorbia pulcherrima Willd. ex Klotzsch) leaves. The percentage contributions of biochemical and molecular composition; structure and ultrastructure; photochemical variables from A–PAR curves; carboxylative CO_2 assimilation from A–Ci curves; chlorophyll a fluorescence – PSII energy partitioning at 1500 µmol photons m^{-2} s^{-1}; JIP-test parameters; and phenomenological energy fluxes per PSII cross-section are shown. For each principal component, the sum of the group contributions equals 100%.

**Groups**	**PC1 (%)**	**PC2 (%)**
Biochemical and molecular composition	17.50	7.17
Structures and ultrastructures	15.04	11.36
Photochemical variables (*A*-PAR)	15.19	9.21
Carboxylative CO_2 assimilation	15.82	5.77
Chlorophyll *a* fluorescence - PSII energy partitioning	20.45	7.22
JIP-test parameters	5.91	30.96
Phenomenological fluxes	10.09	28.31
**TOTAL**	**100**	**100**
